# Both Conifer II and Gnetales are characterized by a high frequency of ancient mitochondrial gene transfer to the nuclear genome

**DOI:** 10.1186/s12915-021-01096-z

**Published:** 2021-07-28

**Authors:** Sheng-Long Kan, Ting-Ting Shen, Jin-Hua Ran, Xiao-Quan Wang

**Affiliations:** 1 State Key Laboratory of Systematic and Evolutionary Botany, Institute of Botany, Chinese Academy of Sciences, Beijing, 100093 China; 2 University of Chinese Academy of Sciences, Beijing, 100049 China; 3 School of Earth Sciences, East China University of Technology, Nanchang, 330013 China

**Keywords:** Mitochondrial gene transfer, Gene content variation, Evolutionary fate, Gymnosperm, Conifer II, Gnetales

## Abstract

**Background:**

Mitochondrial gene transfer/loss is common in land plants, and therefore the fate of missing mitochondrial genes has attracted more and more attention. The gene content of gymnosperm mitochondria varies greatly, supplying a system for studying the evolutionary fate of missing mitochondrial genes.

**Results:**

Here, we studied the tempo and pattern of mitochondrial gene transfer/loss in gymnosperms represented by all 13 families, using high-throughput sequencing of both DNA and cDNA. All 41 mitochondrial protein-coding genes were found in cycads, *Ginkgo* and Pinaceae, whereas multiple mitochondrial genes were absent in Conifer II and Gnetales. In Conifer II, gene transfer from mitochondria to the nucleus followed by loss of the mitochondrial copy was common, but complete loss of a gene in both mitochondrial and nuclear genomes was rare. In contrast, both gene transfer and loss were commonly found in Gnetales. Notably, in Conifer II and Gnetales, the same five mitochondrial genes were transferred to the nuclear genome, and these gene transfer events occurred, respectively, in ancestors of the two lineages. A two-step transfer mechanism (retroprocessing and subsequent DNA-mediated gene transfer) may be responsible for mitochondrial gene transfer in Conifer II and Gnetales. Moreover, the mitochondrial gene content variation is correlated with gene length, GC content, hydrophobicity, and nucleotide substitution rates in land plants.

**Conclusions:**

This study reveals a complete evolutionary scenario for variations of mitochondrial gene transferring in gymnosperms, and the factors responsible for mitochondrial gene content variation in land plants.

**Supplementary Information:**

The online version contains supplementary material available at 10.1186/s12915-021-01096-z.

## Background

Compared with the almost unchanged mitochondrial protein-coding gene content in animals and certain other eukaryotes, the loss of mitochondrial genes frequently occurred in many land plant lineages, with the mitochondrial gene number ranging from 19 (*Viscum scurruloideum*) to > 50 (*Marchantia polymorpha*) [1–6]. It is generally believed that loss of protein-coding genes from the mitogenome may occur following functional transfer of a gene to the nucleus [3, 7–16]. However, the loss of a gene from the mitochondrial compartment does not necessarily imply its functional transfer to the nucleus, particularly for ribosomal protein genes, which were frequently lost in land plants, especially in angiosperms [2, 17–19]. For example, almost all ribosomal protein genes were missing from the mitogenome of *Zostera*, but only a subset of them were found in the nucleus [20]. In addition, a mitochondrial gene may have been replaced by a homologous gene originating from chloroplast or nuclear DNA [21, 22]. Although previous studies have investigated the evolutionary fate of mitochondrial genes transferred to the nuclear genome, most of them explored a single gene or focused on a specific lineage with a relatively short evolutionary history [7, 10, 15, 23]. It is of great interest to investigate the evolutionary dynamics/fates of mitochondrial genes in major clades of land plants with long evolutionary histories.

The gene content of gymnosperm mitochondria shows great variation, especially among different lineages [4, 24–26], providing a good system for studying the evolutionary fate of missing mitochondrial genes. As the sister group of angiosperms, gymnosperms represent four of the five main clades of seed plants with a crown age dated to the Carboniferous, and Conifer II (non-Pinaceae conifers or cupressophytes) has been resolved as a unique lineage in gymnosperms [27, 28]. The complete assembly of plant mitochondrial genomes (mitogenomes) remains challenging due to their complex and variable structures [29]. To date, only six mitogenomes have been sequenced for gymnosperms [25, 26, 30–32], although over 100 angiosperm mitogenomes have been sequenced (https://www.ncbi.nlm.nih.gov/genome/browse#!/organelles/). A comparison of mitochondrial gene content among gymnosperms revealed an evolutionary stasis in *Cycas taitungensis*, *Ginkgo biloba*, and three Pinaceae species (*Pinus taeda*, *Picea abies*, and *Picea sitchensis*), in contrast to extensive gene loss in *Taxus cuspidata* and *Welwitschia mirabilis*. The *Cycas*, *Ginkgo*, and Pinaceae mitogenomes contain 41 protein genes, as in the ancestors of angiosperms [33]. In contrast, *Taxus* and *Welwitschia* have lost not only the *sdh3* gene but also eight and eleven ribosomal protein genes, respectively [25, 26]. By comparing mitochondrial gene and intron contents among 15 diverse gymnosperm species, Guo et al. [24] found that Gnetales and Conifer II mitogenomes underwent extensive gene and intron losses, but they did not investigate the fate of the missing mitochondrial genes, and their study did not sample several phylogenetically important families such as Ephedraceae, Cephalotaxaceae, and Sciadopityaceae. In addition, Kan et al. [26] reported the mitogenome sequence of *Taxus cuspidata* and found that eight genes of this species have been transferred to the nucleus. Therefore, it would be interesting to investigate the evolutionary patterns of mitochondrial genes in gymnosperms and the fate of missing mitochondrial genes in particular, based on a complete sampling at the family level and a joint analysis of both genomic and transcriptomic data.

Various hypotheses have been proposed to explain why frequent gene transfer events occurred in plant mitochondria. Some adaptive hypotheses, such as Muller’s ratchet, genomic streamlining, and avoidance of free radicals, may be plausible mechanisms for promoting transfer from small mitochondrial genomes such as in animals and bacteria [34], except that “beneficial mutations” may play a role in gene transfer events of plant mitochondria [3]. Berg and Kurland [35] proposed a neutral model of gene transfer, which suggested that mitochondrial DNAs were frequently transferred to the nuclear genome, but only certain genes were activated by acquiring presequences and regulatory elements in the nuclear genome, and fixation of beneficial mutations allowed the nuclear copy to outcompete its mitochondrial counterpart [3, 35]. Liu et al. [15] provided a detailed portrayal of structural and sequence evolution for mitochondrial genes transferred to the nucleus by performing comparative analyses of 77 transferred genes in various angiosperms and found that many of them contain mitochondrial targeting presequences and potentially 5′ cis-regulatory elements. In addition, due to that gene transfer/loss is punctuated, why are mitochondrial genes retained in mitochondrial genome? To investigate this question, Johnston and Williams [1] analyzed more than 2000 eukaryotic mitochondrial genomes and found that mitochondrial genes with high GC content and high hydrophobicity are prone to be retained in the mitogenome. Mitochondrial gene transfer/loss is an ongoing process in land plants [1–3]. However, previous studies on mitochondrial gene content variation in land plants focused primarily on angiosperms and earlier land plants [36, 37]. The study of more samples, especially from gymnosperms, is essential to understand why gene content is variable in plant mitochondria.

In this study, we investigated the mitochondrial gene content variation and the fate of missing mitochondrial genes in gymnosperms represented by all 13 families and 19 genera using both DNAs and cDNAs generated from high-throughput sequencing. Then, the mechanisms underlying mitochondrial gene transfer were studied. Moreover, the possible factors related to the variation of mitochondrial gene content were explored based on an analysis of all available data of land plant mitogenomes.

## Results

### Basic information of mitochondrial draft genome assembly

The raw data generated from each species were roughly equal to their genome size. After assembly, 15 to 532 mitochondrial scaffolds were obtained from different samples with average *k-mer* coverage from 6.59 to 180.37. In total, the size of the draft mitogenome ranged from 0.34 to 6.17 Mb with GC contents from 44.08 to 53.20% (Additional file 1: Table S1).

### Mitochondrial gene content variation in gymnosperms

**Fig. 1:**
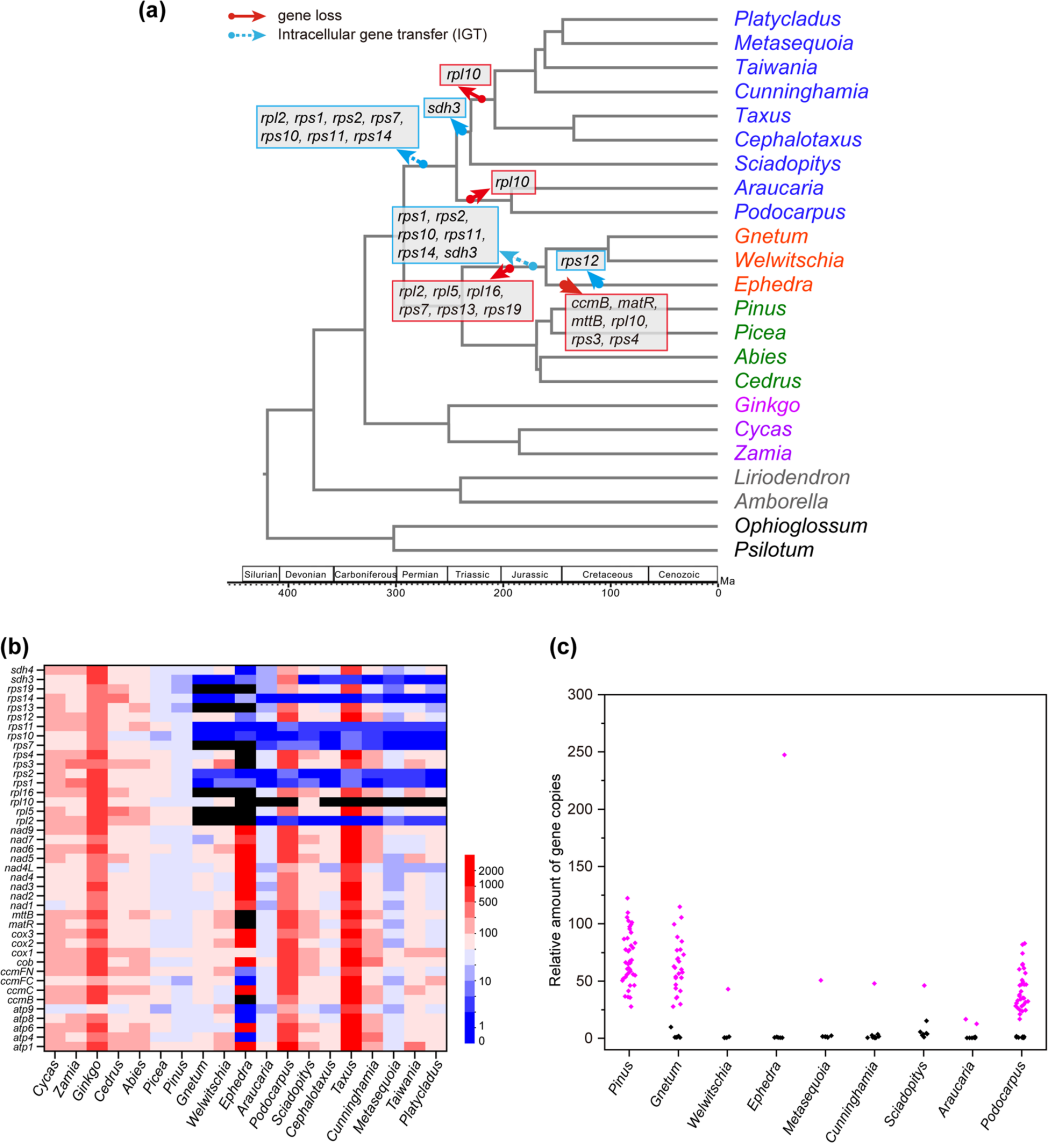
Patterns of loss and transfer of mitochondrial protein-coding genes in gymnosperms with sequencing depth and relative amount of gene copies of these genes. *LEAFY* was used as a reference (**c**) for mitochondrial and transferred genes in gymnosperms. Black color (**b**) indicates gene loss. Purple and black indicate mitochondrial and transferred genes, respectively

We searched the 41 mitochondrial protein-coding genes present in the common ancestor of seed plants [25] from the studied gymnosperm species. All 41 genes were found in cycads (*Cycas revoluta* and *Zamia furfuracea*), *Ginkgo* (*Ginkgo biloba*), and Pinaceae (*Abies firma*, *Cedrus deodara*, *Picea smithiana*, and *Pinus armandii*). In Conifer II, 32 genes were present in all species. Araucariaceae (*Araucaria cunninghamii*) and Podocarpaceae (*Podocarpus macrophyllus*) also contained a *sdh3* gene, and Sciadopityaceae (*Sciadopitys verticillata*) had a *rpl10* gene. Notably, many fewer mitochondrial protein-coding genes occurred in Gnetales. *Gnetum montanum* and *Welwitschia mirabilis* contain 29 genes, whereas *E*. *przewalskii* has only 22 genes. It is interesting that intact or partial sequences of an additional 6–8 homologs of mitochondrial genes were exclusively found in the transcriptomic and/or genomic data of Conifer II and Gnetales (Fig. 1a and Additional file 2: Table S2), which were inferred to be lost in certain species of Conifer II, and *Welwitschia* and *Gnetum* among Gnetales in previous studies [24–26].

To identify whether these homologs have been transferred to the corresponding species’ nuclear genome, depth of sequencing coverage and real-time PCR were applied in this study. Analysis of average sequencing depth showed that these genes have the same sequencing depth as the single-copy nuclear gene *LEAFY* in Conifer II and Gnetales (Fig. 1b and Additional file 3 Figure S1). In addition, the real-time PCR experiments confirmed that the relative amounts of gene copies of these genes were consistent with *LEAFY*, less than those of the mitochondrial genes (Fig. 1c).

### Gene structure of mitochondrial and putative transferred genes

**Fig. 2:**
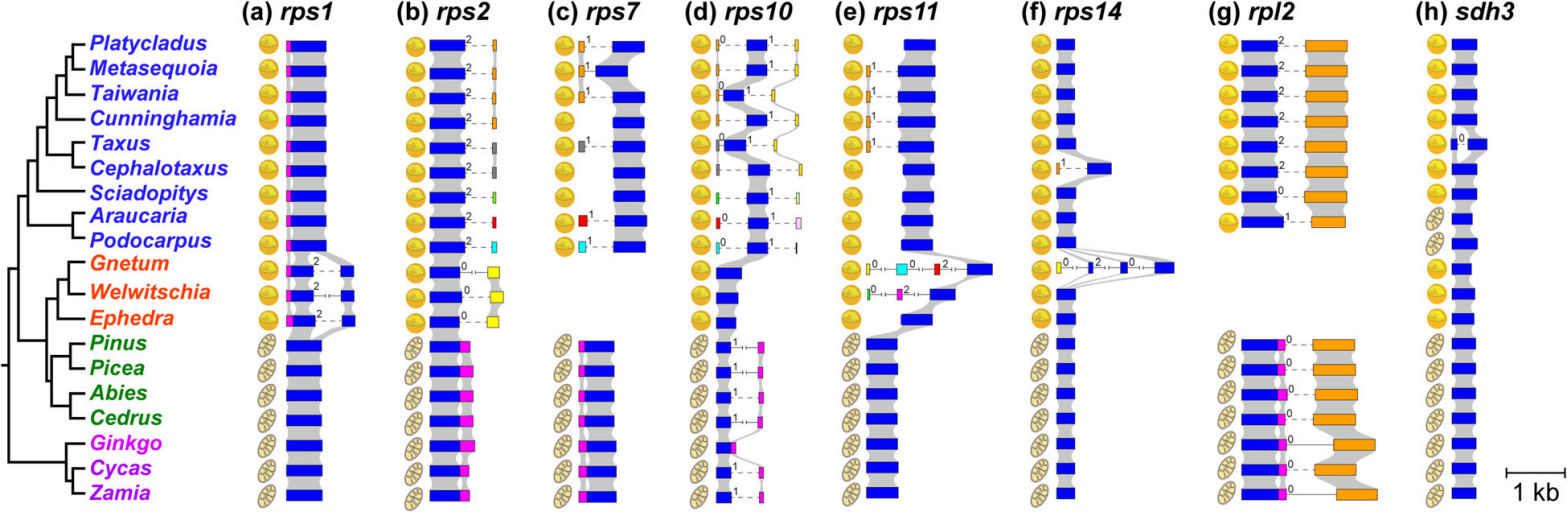
Structure of eight transferred genes and their mitochondrial homologs in gymnosperms. Exons are indicated with boxes, and microsyntenic relationships between orthologous genes are shown with gray shadows. Introns are indicated with lines; the dashed lines indicate that the intron lengths are uncertain and the cross lines indicate that the intron lengths are too long to be shown. Numbers between boxes and lines represent phases of intron. **a** *rps1*, **b** *rps2*, **c** *rps7*, **d** *rps10*, **e** *rps11*, **f** *rps14*, **g** *rpl2*, and **h** *sdh3*

The distribution pattern of introns in the gymnosperm mitochondrial genes was similar to that reported in Guo et al. [24]. In brief, the mitogenomes of two cycads contained 21 *cis*- and five *trans*-spliced introns. Compared to those of the cycads, the mitogenome of *Ginkgo* lost only one intron (rps10i235). In Pinaceae, all 26 introns were found, but eight of them were converted from *cis*- to *trans*-spliced. In the mitogenomes of Conifer II, 14–15 introns were found, of which six to seven changed from *cis*- to *trans*-spliced. Compared with cycads, 11–12 introns were lost in Conifer II, of which two were lost due to gene loss. In the mitogenomes of Gnetales, only ten introns were found in *Welwitschia*, of which two showed changes in the splicing mode. In the lost 16 introns, two were lost with genes. In contrast, *Gnetum* contains 22 introns, and only two introns displayed a changed spliced mode. Most surprisingly, 1 (nad2i542) *cis*- and 17 *trans*-spliced introns were found in *Ephedra*. In addition, it is uncertain whether nad5i1477 and nad5i1872 existed in *Ephedra* (Additional file 4: Table S3). In the putative transferred genes, the *rpl2* gene contains one intron in all gymnosperm lineages excluding Gnetales (no homolog was found in gnetophytes), but this intron has different phases in Conifer II and cycads+*Ginkgo*+Pinaceae. In the remaining genes, intron gain was found in five genes (*rps1*, *rps2*, *rps11*, *rps14*, and *sdh3*), whereas intron loss occurred only in *rps10* in Gnetales. One intron of *rps2* was gained in both Conifer II and Gnetales but with different phases, and one intron of *sdh3* was found in *Taxus*. For *rps11*, *Gnetum* and *Welwitschia* obtained three and two introns, respectively, whereas no intron was found in *Ephedra*. One and three introns were found only in *rps14* of *Cephalotaxus* and *Gnetum*, respectively (Fig. 2).

The presequence of some putative transferred genes derived from another nuclear gene encoding a mitochondrial protein. For example, the presequence of *sdh3* encodes a mitochondrial chaperonin heat shock protein in Cupressaceae and Cephalotaxaceae. In addition, some putative transferred genes have a presequence acquired from a nuclear gene encoding a nonmitochondrial protein or from an unknown source or have no presequence (Additional file 5: Table S4).

### Variation of RNA editing sites in the mitochondrial and putative transferred genes

Due to the different expression levels of mitochondrial genes at different developmental periods of plants [38], it is difficult to identify the exact number of RNA editing sites of mitochondrial genes. In this study, RNA editing sites were found in all 17 species except *Welwitschia*, although few RNA editing sites were identified in *Welwitschia* in a previous study of Fan et al. [39] (Additional file 6: Table S5). In addition, we compared the RNA editing pattern of eight putative transferred genes (*rps1*, *rps2*, *rps7*, *rps10*, *rps11*, *rps14*, *rpl2*, and *sdh3*) in gymnosperms. No RNA editing sites were found in the putative transferred genes of Conifer II and Gnetales, and a majority of sites corresponding to the RNA editing sites of their mitochondrial homologous genes in Pinaceae, cycads, and *Ginkgo* were changed from C to T in Conifer II and Gnetales (Additional file 7: Figure S2).

### Phylogenetic analysis and ancestral state reconstruction

The phylogenetic analyses of gymnosperms based on mitochondrial protein-coding genes supported the “Gnepine” hypothesis that Gnetales and Pinaceae are sister groups, whereas the phylogenetic relationship constructed using the putative transferred genes and their homologs supported the “GneCup” hypothesis (Gnetales sister to Conifer II) (Additional file 8: Figure S3). The single-gene trees of the putative transferred genes and their homologs showed different topologies. In the *rps2*, *rps10* and *rps14* gene trees, Conifer II and Gnetales were clustered together, whereas in the *rps1* and *rps11* gene trees, these two lineages did not form a monophyletic group (Additional file 9: Figure S4).

The result of the ancestral state reconstruction indicated that complicated transfer and loss events of mitochondrial genes occurred in gymnosperms. For example, *rps1*, *rps2*, *rps10*, *rps11*, and *rps14* may have undergone intracellular transfer events in ancestors of Conifer II and Gnetales, respectively, while *rpl2* and *rps7* were transferred to the nucleus in the ancestor of Conifer II but lost in the common ancestor of Gnetales. Transfer of *sdh3* may have occurred in Conifer II, excluding *Araucaria* and *Podocarpus*, and the common ancestor of Gnetales (Additional file 10: Figure S5). In addition, in Conifer II, the mitochondrial *rpl10* gene was only found in *Sciadopitys*. Furthermore, *rpl5*, *rpl16*, *rps13*, and *rps19* were lost in the common ancestor of Gnetales, and *ccmB*, *matR*, *mttB*, *rpl10*, *rps3*, and *rps4* were not found in *Ephedra* (Fig. 1a).

### Variation of evolutionary rates between mitochondrial and putative transferred genes

**Fig. 3:**
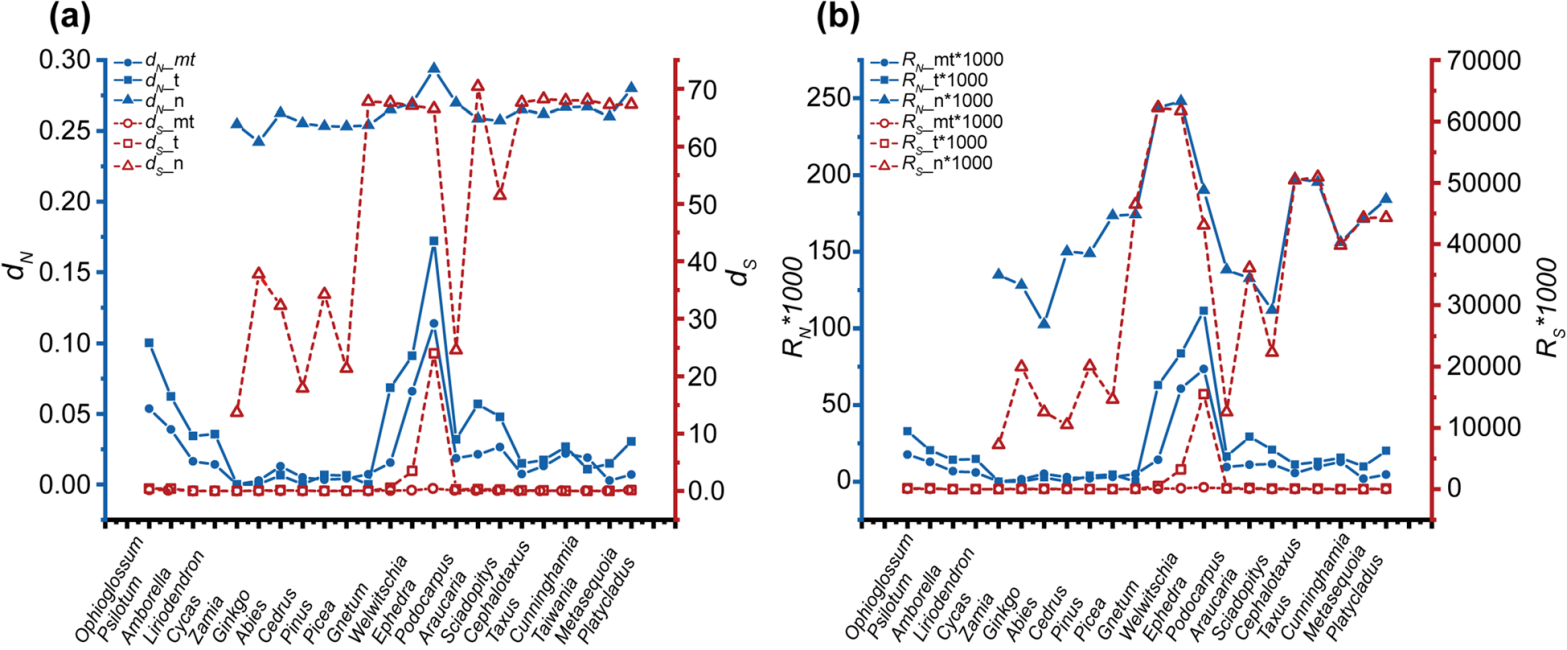
Sequence variation parameters of mitochondrial and transferred genes for each taxon using the *LEAFY* gene as a control. **a** *d*_*N*_ and *d*_*S*_. **b** *R*_*N*_ and *R*_*S*_. mt, that the mitochondrial genes did not transfer to the nuclear genome in all taxa; t, the transferred genes in Conifer II and Gnetales and their mitochondrial homologs in other gymnosperms; n, the *LEAFY* gene

The synonymous and nonsynonymous substitution rates (*d*_*S*_ and *d*_*N*_) of mitochondrial protein-coding genes and putative transferred genes were significantly different among different lineages of gymnosperms. All genes of cycads, *Ginkgo*, Pinaceae, and Conifer II have lower synonymous substitution rates than Gnetales, and nonsynonymous substitution rates are lower in cycads, *Ginkgo*, and Pinaceae than in Conifer II and Gnetales. In addition, the rates of synonymous and nonsynonymous substitutions of putative transferred genes significantly increased (Fig. 3a). The pattern of absolute substitution rate of mitochondrial protein-coding genes is consistent with the relative substitution rate. The absolute synonymous substitution rates of putative transferred genes were significantly accelerated in Conifer II and Gnetales, and the absolute nonsynonymous substitution rates were only accelerated in Gnetales (Fig. 3b). Furthermore, the evolutionary rates of both mitochondrial and putative transferred genes were lower than that of the nuclear gene *LEAFY* (Fig. 3).

### GC content and hydrophilicity of mitochondrial and putative transferred genes

**Fig. 4:**
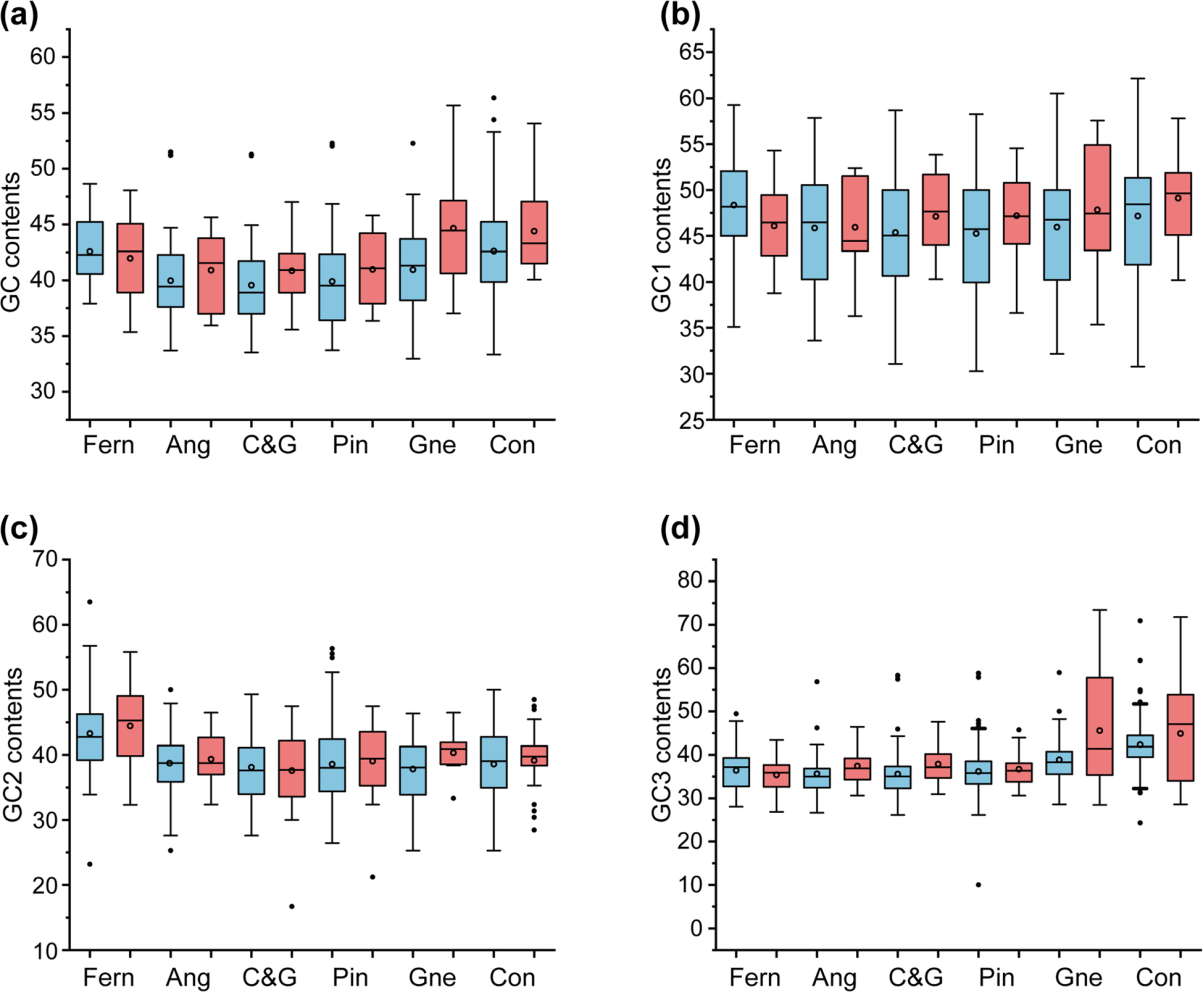
GC content variation in the genes of the sampled species. **a** All positions of the codon, **b** first position of the codon, **c** second position of the codon, and **d** third position of the codon. Ang, angiosperms; C&G, Cycads and *Ginkgo*; Pin, Pinaceae; Gne, Gnetales; Con, Conifer II. Blue indicates the transferred genes in Conifer II and Gnetales and their mitochondrial homologs in other taxa, and red indicates the conserved mitochondrial genes

The GC contents of mitochondrial protein-coding and putative transferred genes in different lineages of gymnosperms were compared. For the mitochondrial protein-coding genes, the GC2 contents were similar in different lineages, and the GC and GC3 contents were higher in Conifer II and Gnetales than in cycads, *Ginkgo* and Pinaceae. For the putative transferred genes, the GC, GC2 and GC3 contents were significantly higher than those of mitochondrial genes in Conifer II and Gnetales, as well as their homologous genes in the mitochondrial genome of cycads, *Ginkgo* and Pinaceae (Fig. 4).

**Fig. 5:**
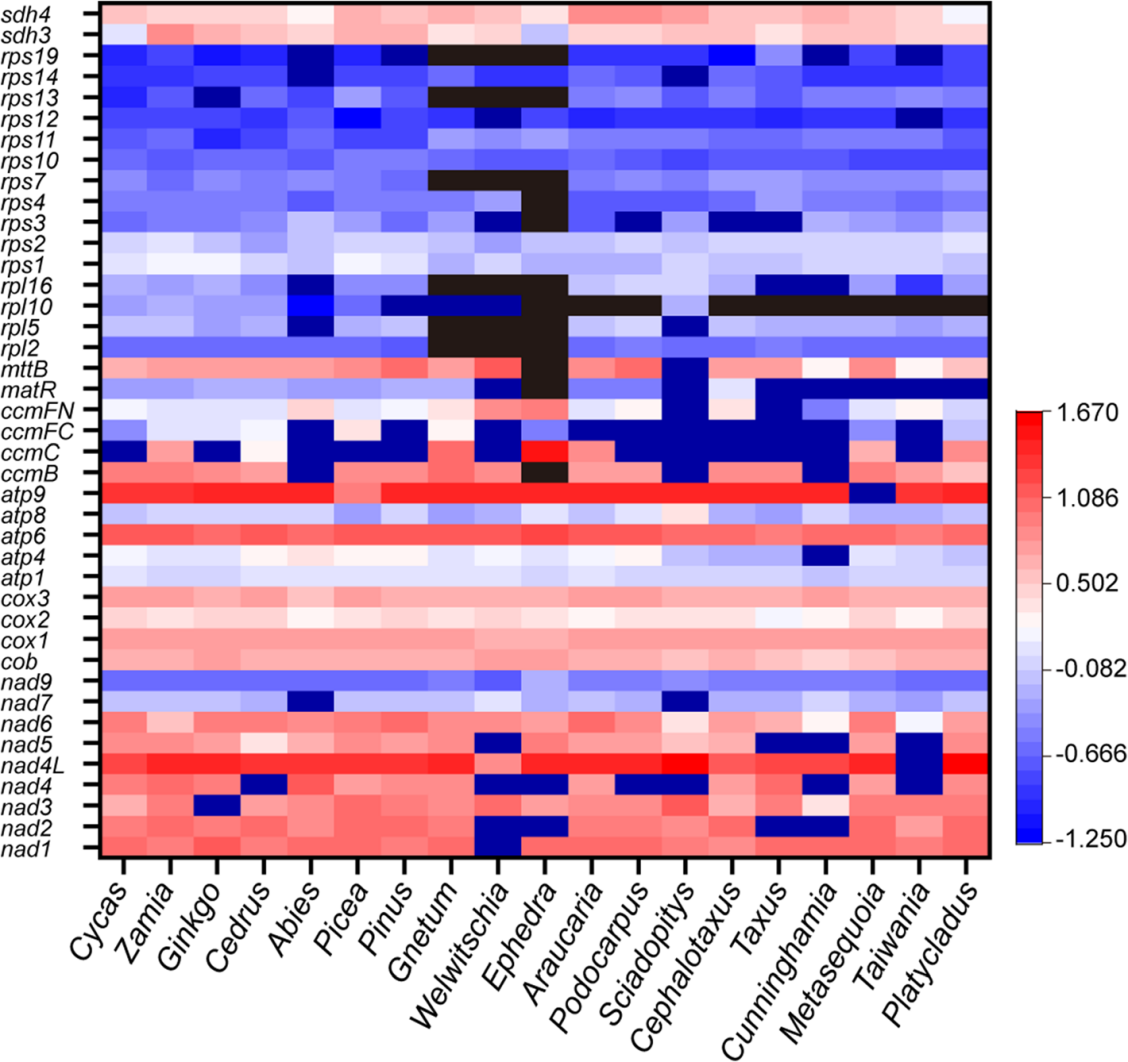
Grand average of hydropathicity (GRAVY) of mitochondrial genes and their transferred homologs in gymnosperms. Black indicates gene loss

All putative transferred genes (except *sdh3*) encode hydrophilic proteins. In addition, hydrophilic mitochondrial protein-coding genes are more common in Conifer II and Gnetales than in cycads, *Ginkgo* and Pinaceae (Fig. 5).

### Factors influencing mitochondrial gene content in land plants

To test the factors that can influence the mitochondrial gene content, we downloaded almost all published mitogenomes of land plants from NCBI and calculated the gene length, GC content, *d*_*N*_ and *d*_*S*_ values, and hydrophilicity for all the downloaded genes. The results showed that mitochondrial genes with longer length, higher GC content and stronger hydrophobicity were more likely to be preserved in the mitogenome (Additional file 11: Figure S6a,b,c). The number of mitochondrial genes has a notably weak, moderate, and weak correlation with GC content (*R*^*2*^_(*GC*)_ = 0.0300) (Additional file 11: Figure S6d), synonymous substitution rate (*R*^*2*^_(*dS*)_ = 0.3467), and nonsynonymous substitution rate (*R*^*2*^_(*dN*)_ = 0.1079) (Additional file 11: Figure S6e,f), respectively.

## Discussion

### A high frequency of ancient mitochondrial gene transfer to the nucleus was found in both Conifer II and Gnetales

Based on analyses of both DNA and cDNA sequences generated from high-throughput sequencing, we investigated the variation of mitochondrial gene contents and the fate of missing mitochondrial genes by sampling representative species from all families of gymnosperms. Although a few fast-evolving mitochondrial genes might be difficult to identify from the draft genomes, our results should be reliable when compared with the published mitogenomes of gymnosperms and the study of Guo et al. [24]. Similar to previous studies, all 41 mitochondrial protein-coding genes were found in cycads (*Cycas* and *Zamia*), *Ginkgo* and Pinaceae (*Abies*, *Cedrus*, *Pinus* and *Picea*), whereas many were not found in the mitogenomes of Conifer II or Gnetales [24–26, 30–32]. Notably, we found that gene transfer was common but that gene loss was rare in Conifer II, whereas both gene transfer and loss commonly occurred in Gnetales (Fig. 1a). For example, in the ancestor of Conifer II, seven mitochondrial genes (*rpl2*, *rps1*, *rps2*, *rps7*, *rps10*, *rps11*, and *rps14*) were transferred to the nucleus, but only *rpl10* was lost in the descendants. In contrast, six genes were lost and another six genes were transferred to the nuclear genome in the ancestor of Gnetales, followed by the additional loss of six genes (*ccmB*, *matR*, *mttB*, *rpl10*, *rps3*, and *rps4*) and intracellular transfer of one gene (*rps12*) in *Ephedra* (Fig. 1a).

Interestingly, five of the genes that have been transferred to the nuclear genome are shared between Conifer II and gnetophytes, including *rps1*, *rps2*, *rps10*, *rps11*, and *rps14*. This phenomenon, together with the fact that the phylogenetic tree inferred from these five genes suggests a sister relationship between Conifer II and gnetophytes (Additional file 8: Figure S3), seems to support the Gnecup hypothesis. However, the nucleotide substitution rates of transferred genes in Conifer II and gnetophytes are much higher than those of their homologs in cycads, ginkgo, and Pinaceae, which may have led to long-branch attraction in phylogenetic reconstruction [40, 41]. Although the evolutionary rates of putative transferred genes were still lower than that of the *LEAFY* gene, it could be because these genes had evolved under functional constraints from mitochondria after transfer to the nucleus. In addition, the Gnepine hypothesis is highly supported by the phylogenetic tree inferred from the mitochondrial protein-coding genes. Although One Thousand Plant Transcriptomes Initiative [42] reported that the placement of Gnetales conflicted among the ASTRAL, supermatrix, and plastome-based trees and both Gnecup and Gnepine hypotheses were supported by the calculation of gene-tree quartet frequencies; Ran et al. [28] reconstructed a robust phylogeny of seed plants based on 1308 nuclear genes, supporting the Gnepine hypothesis. Therefore, it is very likely that the five genes were transferred to the nuclear genome in the ancestors of Conifer II and Gnetales, respectively. This inference is also supported by the ancestral state reconstruction of gene transfer/loss events (Additional file 10: Figure S5). It is interesting that the presequence of *sdh3* encodes a mitochondrial chaperonin heat shock protein in Cupressaceae and Cephalotaxaceae, supporting the occurrence of an ancient gene transfer event (Additional file 5: Table S4). However, the presequences of most putative transferred genes are diverse, which could have resulted from separate gene activations or from extensive recombination events in different lineages after a single ancient gene activation following transfer to the nucleus [15].

Our analysis of mitochondrial gene content variation indicates that the basal groups in both gymnosperms and angiosperms encode almost the complete set of mitochondrial protein-coding genes, similar to the common ancestor of seed plants [25, 30, 33, 43], and genes encoding small and large subunit ribosomal proteins and succinic acid dehydrogenase are more prone to be transferred/lost (Fig. 1a) [2–4, 8, 9, 20, 21]. However, during the evolution of angiosperms, gene transfer/loss events generally occurred in a genus or even in a species except for a few genes [2, 3, 8, 9, 12, 20, 21], whereas in gymnosperms, except for *Ephedra*, most of the mitochondrial gene transfer/loss events occurred in the common ancestors of Conifer II and Gnetales, respectively (Fig. 1a).

### The two-step transfer mechanism may be the method of mitochondrial gene transfer in Conifer II and Gnetales

During plant evolution, the phenomenon of mitochondrial gene transfer to the nuclear genome is very common, but the mechanism of intracellular gene transfer is still controversial [13, 44–46]. Three main mechanisms were proposed for intracellular gene transfer in plants: direct DNA-mediated, direct RNA-mediated, and two-step transfer mechanisms (retroprocessing and subsequent DNA-mediated gene transfer) [13, 45]. If these DNAs were directly transferred from organellar DNA to the nuclear genome, then gene introns with the same phases and positions and RNA editing sites similar to their mitochondrial homologs can be found. Theoretically, the existence of RNA editing sites and group II introns in mitochondrial genes would impede the expression of transferred genes. In this study, the transferred *rpl2* gene lost the mitochondrial intron in all taxa of Conifer II (Fig. 2). In addition, in the transferred *rps1*, *rps2*, *rps10*, *rps11*, *rps14* and *rpl2* genes, most RNA editing sites found in cycads, *Ginkgo*, and Pinaceae were converted from C to T in Conifer II and Gnetales (Additional file 7: Figure S2). Therefore, these genes could have been transferred via a direct RNA-mediated mechanism or the two-step transfer mechanism. However, previous studies have shown that direct transfer of organelle DNA to the nuclear genome is notably frequent, while direct transfer of organelle RNA to the nuclear genome is quite rare [47, 48]. Moreover, Ran et al. [49] found that the *rps3* gene underwent a “retroprocessing” event in Conifer II, resulting in the loss of introns and RNA editing sites, and thus may represent an initial stage of gene transfer. Considering the above information, coupled with the fact that the mitochondrial introns and RNA editing sites were lost in Conifer II and Gnetales, we deduce that retroprocessing and the following DNA-mediated gene transfer pathway may be responsible for mitochondrial gene transfer in Conifer II and Gnetales.

In addition to the counterparts encoded by the nuclear genome, the homologs of chloroplast genes or chloroplast-derived genes encoded by the nuclear genome can also function in mitochondria [2, 21, 22, 46]. In Conifer II and Gnetales, certain genes were not found in either the mitochondria or the nuclear genome. For example, in Conifer II, the *rpl10* gene only exists in the mitochondrial genome of *Sciadopitys*, and in Gnetales, six genes (*rpl2*, *rpl5*, *rpl16*, *rps7*, *rps13*, and *rps19*) were lost in the common ancestor of Gnetales, and another six genes (*ccmB*, *matR*, *mttB*, *rpl10*, *rps3*, and *rps4*) were lost from the mitogenome of *Ephedra*. Considering that most of the above genes participate in important physiological processes such as protein synthesis and energy metabolism [25, 50], these genes might have been functionally replaced by chloroplast genes or cytosol-derived genes encoded in the nuclear genome [21, 22]. However, recent studies have found that a larger number of mitochondrial genes (e.g., *nad1*, *nad2*, *nad3*, *nad4*, *nad4L*, and *nad5*) were lost in select angiosperms, such as *Viscum* (Viscaceae) [5, 17, 19], and hence, we cannot rule out the possibility that certain mitochondrial genes of Gnetales have been lost directly.

It is intriguing that the *matR* gene was not found in the mitochondrial genome of *Ephedra* (Fig. 1a). The mitochondrial *matR* gene has a conserved domain with mature enzyme activity and a degenerated domain with reverse transcriptional activity involved in the splicing of mitochondrial group II introns [51]. Generally, the *matR* gene of seed plants is located in the fourth intron of *nad1* (nad1i728) [11, 25, 30]. Nevertheless, the *matR* gene was lost in certain angiosperms, such as Malpighiales (*Croizatia brevipetiolata* and *Lachnostylis bilocularis*) and Viscaceae (*Viscum* and *Phoradendron*) [5, 17, 19, 52]. Currently, it is unclear why these plants do not need the *matR* gene and what effect may result from the loss of *matR*. Grewe et al. [11] found that *matR* was transferred to the nuclear genome and split into two genes with respective reverse transcriptional activity and mature enzyme activity in *Pelargonium*. In addition, the nuclear genome can encode four mature enzymes that are transported to the mitochondria, such as nMAT1 participating in the *trans*-splicing of nad1i394 in *Arabidopsis* [51]. Furthermore, among the few hundreds of currently available mitogenome sequences, there is no loss of *mttB* and the loss of *ccmB* only occurred in *Viscum scurruloideum* [4, 5]. Therefore, in *Ephedra*, the *matR*, *mttB*, and *ccmB* genes could also have been transferred to the nuclear genome, but we did not find their nuclear homologs due to great sequence divergence, although it is possible that these genes have been completely lost.

### Several factors may be related to mitochondrial gene content variations in land plants

In gymnosperms, almost all gene transfer/loss events are notably ancient, and it is difficult to know why a large number of mitochondrial genes were transferred/lost hundreds of millions of years ago, but the gene content of the mitochondrial genome has remained stable in the later period. Therefore, the gene content variation in gymnosperms could be more likely to be related to the question “why mitochondrial genes are retained in mitochondrial genome”. Based on the newly generated data from a complete sampling of gymnosperm families in combination with plant mitochondrial genomes in public databases, we conducted a comparative analysis to find the factors that might influence mitochondrial gene content variation in land plants and obtained the following findings. First, the easily transferred genes are generally small and hydrophilic with low GC content, supporting the hypothesis that relatively small, low GC content, and soluble proteins such as ribosomal proteins can be easily transported from the nucleus to mitochondria (Additional file 11: Figure S6a,b,c) [1, 2]. Second, in land plants, the higher the GC content, the fewer the mitochondrial genes, implying that mitochondria with high GC content contain fewer genes (Additional file 11: Figure S6d). Third, more mitochondrial genes can be transferred or lost when the nucleotide substitution rates of extant mitochondrial genes are high (Additional file 11: Figure S6e,f). The synonymous and nonsynonymous substitutions of mitochondrial genes are higher in Conifer II and Gnetales than in cycads, *Ginkgo* and Pinaceae (Fig. 3). In conclusion, four factors, including gene length, GC content, hydrophobicity, and nucleotide substitution rates, may be related to mitochondrial gene content variation in land plants.

## Conclusions

In this study, we investigated the variation of mitochondrial gene contents and the fate of missing mitochondrial genes by an integrated analysis of the high-throughput sequencing data of DNA and cDNA of representative species from all 13 families and 19 genera of gymnosperms. We found a high frequency of ancient mitochondrial gene transfer to the nucleus in both Conifer II and Gnetales and deduced that retroprocessing followed by DNA-mediated gene transfer could be responsible for mitochondrial gene transfer in Conifer II and Gnetales based on the fact that the mitochondrial introns and RNA editing sites were lost in transferred genes. In addition, we explored the possible factors related to the variation of mitochondrial gene content in land plants based on a combined analysis of the data generated in the present study that cover all gymnosperm families and the available plant mitochondrial genome sequences.

## Methods

### Taxon sampling, DNA extraction, and sequencing

A total of 19 species representing all families of gymnosperms were sampled. The high-throughput sequences of both DNA and cDNA of *Taxus cuspidata* were taken from Kan et al. [26]. In addition, the transcriptional and DNA data of the other 18 samples were downloaded from NCBI [28, 53] and sequenced in this study. For high-throughput DNA sequencing, leaf buds were collected for DNA extraction using a modified CTAB (cetyltrimethylammonium bromide) procedure Porebski et al. [54]. Total DNA was sonicated using the Covaris M220, and DNA fragments 500–600 bp in length were purified using the TIANgel Midi purification kit (Tiangen, Beijing, China). A sequencing library was constructed using the NEBNext® Ultra ™ DNA Library Prep Kit for Illumina® (New England Biolabs Inc.), according to the manufacturer’s introductions, and sequenced on an Illumina HiSeq 2500 instrument using the 250 bp paired-end protocol. Additionally, because the mitochondrial genome of *Ephedra przewalskii* was difficult to assemble, we also used long-read sequencing (Oxford Nanopore) technology, following the protocol of Kan et al. [26]. Detailed information is shown in Table S6 (Additional file 12).

### Sequence assembly and mitochondrial gene identification

Due to difficulties in the complete assembly of plant mitogenomes [29], we did not try to assemble all the mitochondrial genes into a single contig. Instead, we used the known mitochondrial genes of gymnosperms as queries to retrieve their homologs from all samples using TBLASTN [55]. The raw reads were trimmed and filtered by Trimmomatic [56] and assembled by SOAPdenovo2 [57] and SPAdes v 3.13.2 [58]. The assembly of the *E*. *przewalskii* mitochondrial genome was referred to Kan et al. [26]. All mitochondrial genes were retrieved from the assembled contigs and transcriptomes using the mitochondrial genes of *Cycas taitungensis* [30], *Pinus strobus*, and *Araucaria heterophylla* as queries. If one gene was not found in the DNA or transcriptome databases of *Gnetum* and *Taxus* obtained in this study, we first searched it in the published genome databases and then designed multiple primer pairs to amplify it from total DNA or RNA [59, 60]. If one gene was not found in the other species, we amplified it from total DNA or RNA. Total RNA extraction, purification, and first-strand cDNA synthesis were performed following the protocols of Ran et al. [49]. The primers are shown in Table S7 (Additional file 13).

### Identification of genes transferred to the nuclear genome and mitochondrial scaffolds

Similar to Kan et al. [26], we used two methods, i.e., depth of sequencing coverage and real-time PCR, to identify mitochondrial genes that have been transferred to the nuclear genome. In the real-time PCR analysis, single-copy nuclear gene *LEAFY* was used as an experimental control. All putative mitochondrial and transferred genes were amplified in three species, *Pinus armandii*, *Gnetum montanum*, and *Podocarpus macrophyllus*, representing Pinaceae, Gnetales and Conifer II, respectively, and we found that the results were the same as that generated by using the depth of sequencing coverage. Therefore, we only used *nad2* or *nad5* as the mitochondrial gene control to identify the putative transferred genes inferred from the depth of sequencing coverage in other species. The primers are also listed in Table S7 (Additional file 13).

If a scaffold contains identified mitochondrial genes or introns, it is considered to be a mitochondrial scaffold, and its average GC content and *k-mer* coverage are used as the criteria for screening the mitochondrial scaffolds that do not encode mitochondrial genes [24]. The mitochondrial scaffolds were annotated and deposited in GenBank (MW354079–MW354511).

### Identification of the RNA editing sites and analysis of gene structure variations

The DNA and corresponding cDNA sequences of each gene were compared for each species to identify the RNA editing sites in the coding regions. In addition, we identified the positions and number of introns of each gene by comparing the assembled DNA and cDNA sequences. According to the criteria proposed by Guo et al. [24], the splicing mode of mitochondrial intron was determined. Multiple primer pairs were further designed for the determination if the splicing mode of an intron cannot be identified. The cDNA sequence was first amplified to verify the reliability and amplification efficiency of the primer pairs, and then the primer pair with the highest amplification efficiency was used to amplify the DNA sequence to confirm the splicing mode of the intron Table S7 (Additional file 13). In addition, the published genome data of *Taxus chinensis* and *Gnetum montanum* were also used to determine structure of the transferred genes [59, 60]. Moreover, we used BLAST to annotate the N-terminal presequences of putative transferred genes in gymnosperms.

### Evolutionary rate variations between mitochondrial and transferred genes

The evolutionary rate variations between mitochondrial and transferred genes were compared using the *LEAFY* gene as a control. To avoid the influence of RNA editing sites, cDNA sequences were used in the phylogenetic analysis. Two basal angiosperms with all 41 mitochondrial genes, i.e., *Amborella trichopoda* and *Liriodendron tulipifera*, were selected to represent angiosperms [33, 61], and two ferns, i.e., *Ophioglossum californicum* and *Psilotum nudum*, were selected as outgroups [62]. All mitochondrial genes were concatenated directly. If one gene was proven to be transferred to the nucleus in a species, we first reconstructed the single-gene trees so that we could infer when and how many times this gene was transferred to the nuclear genome. The GTRGAMMA and PROTGAMMAAUTO models were used in the nucleotide and AA matrices, respectively, and RaxML v. 8.2.12 was used to reconstruct the phylogenetic relationships with 100 bootstrap replicates [63].

The nucleotide substitution rates (*d*_*S*_ and *d*_*N*_) of each gene were calculated using PAML 4.9e [64]. Absolute rates of substitutions per branch (*R*_*S*_ and *R*_*N*_) were calculated by dividing the nucleotide substitution rates by their divergence times. The divergence times of the seed plants were obtained from Ran et al. [28] and Ran et al. [53], the Angiosperm Phylogeny Website (http://www.mobot.org/mobot/research/apweb/) and the TimeTree web service (http://www.timetree.org/).

### GC content calculation and hydrophobicity prediction

We used a python script to separately calculate the GC content of conserved mitochondrial genes and the transferred genes in Conifer II and Gnetales and their mitochondrial homologs in other gymnosperms in all lineages (ferns, Angiosperms, cycads, *Ginkgo*, Pinaceae, Gnetales, and Conifer II). The hydrophobicity of each protein in each species was predicted using the ProtParam Tool (https://web.expasy.org/protparam/).

## Supplementary Information

**Additional file 1:** **Table S1**. Statistics of mitochondrial assembly.

**Additional file 2:** **Table S2**. Statistics of mitochondrial protein-coding genes in gymnosperms.

**Additional file 3:** **Figure S1**. The sequencing depth of transferred and mitochondrial genes in gymnosperms. Purple and black indicate mitochondrial and transferred genes, respectively.

**Additional file 4:** **Table S3**. Statistics of mitochondrial introns in gymnosperms.

**Additional file 5:** **Table S4**. Annotation of the N-terminal presequences of putative transferred genes in gymnosperms.

**Additional file 6:** **Table S5**. Statistics of RNA editing sites in mitochondrial genome of gymnosperms.

**Additional file 7:** **Figure S2**. Localization of RNA editing sites of eight mitochondrial genes and the corresponding bases of their transferred homologs. The red vertical line indicates that the site was edited in the mitochondrial gene, the blue vertical line indicates that the site was converted from C to T in the DNA sequence, and the black vertical line indicates that the site was C in the DNA sequence and was not edited in the RNA sequence. a, *rps1*; b, *rps2*; c, *rps7*; d, *rps10*; e, *rps11*; f, *rps14*; g, *rpl2*; h, *sdh3*.)

**Additional file 8:** **Figure S3**. Phylogenetic relationships reconstructed by 22 mitochondrial genes (**a**) and 5 transferred genes and their mitochondrial homologs (**b**), respectively.

**Additional file 9:** **Figure S4**. Single-gene tree reconstructed using transferred genes and their mitochondrial homologs.

**Additional file 10:** **Figure S5**. Ancestral state reconstruction of mitochondrial gene transfer/loss in gymnosperms. Yellow circle indicates lost gene, red circle indicates mitochondrial gene, and black circle indicates transferred gene.

**Additional file 11:** **Figure S6**. Correlation between the number of species that preserved the gene in land plants and gene length (**a**), GC content (**b**) and hydrophobicity (**c**) and correlation between number of mitochondrial genes in terrestrial plants and GC content (**d**) and substitution rate (**e** and **f**). *r* indicates the Pearson’s correlation coefficient, and *R*^*2*^ indicates the coefficient of determination in linear regression analysis.

**Additional file 12:** **Table S6**. Samples and data used in this study.

**Additional file 13:** **Table S7**. Primers used in this study.

## Data Availability

Raw sequence data is available through the NCBI SRA under BioProject accession PRJNA665158 [65]. The annotated mitochondrial contigs are deposited in the GenBank under accessions MW354079-MW354511. The alignment files used for analyses are available from the Dryad Digital Repository (10.5061/dryad.98sf7m0hg) [66].

## Abbreviations

NCBI: National Center for Biotechnology Information
*d*_*S*_ and * d*_*N*_: Synonymous and nonsynonymous substitution rates
*R*_*S *_ and * R*_*N*_: Absolute synonymous and nonsynonymous substitution rates
