# Supplementary material for: Both Conifer II and Gnetales are characterized by a high frequency of ancient mitochondrial gene transfer to the nuclear genome
Source: BMC Biol. 2021 Jul 28;19:146. doi: 10.1186/s12915-021-01096-z (PMC8317393; doi:10.1186/s12915-021-01096-z)
Supplement: Supplementary file 1 — Additional file 1: Table S1. Statistics of mitochondrial assembly. [file 12915_2021_1096_MOESM1_ESM.docx]

**Additional file 1: Table S1. Statistics of mitochondrial assembly**

| **Species** | **Kmers** | **Mito. Scaffolds** | **Scaffolds Len.(Mb)** | **GC%** | **Kmer Cov.** |
| --- | --- | --- | --- | --- | --- |
| *Cycas revoluta* | 21,33,55,77,99,127 | 52 | 0.41 | 46.72 | 34.46 |
| *Zamia furfuracea* | 21,33,55,77,99,127 | 136 | 0.62 | 46.43 | 22.20 |
| *Ginkgo biloba* | 21,33,55,77,99,127 | 30 | 0.36 | 50.30 | 96.61 |
| *Pinus armandii* | 21,33,55,77,99,127 | 370 | 3.85 | 45.47 | 6.59 |
| *Picea smithiana* | 21,33,55,77,99,127 | 532 | 6.17 | 44.08 | 9.00 |
| *Abies firma* | 21,33,55,77,99,127 | 172 | 1.33 | 45.78 | 45.01 |
| *Cedrus deodara* | 21,33,55,77,99,127 | 91 | 5.13 | 45.06 | 31.73 |
| *Platycladus orientalis* | 21,33,55,77,99,127 | 79 | 2.46 | 50.64 | 13.69 |
| *Metasequoia glyptostroboides* | 21,33,55,77,99,127 | 248 | 1.77 | 52.17 | 6.82 |
| *Cunninghamia lanceolata* | 21,33,55,77,99,127 | 134 | 1.89 | 51.84 | 26.75 |
| *Taiwania cryptomerioides* | 21,33,55,77,99,127 | 124 | 2.80 | 51.08 | 28.27 |
| *Cephalotaxus sinensis* | 21,33,55,77,99,127 | 118 | 0.34 | 45.32 | 12.72 |
| *Sciadopitys verticillata* | 21,33,55,77,99,127 | 226 | 1.87 | 44.88 | 35.54 |
| *Araucaria cunninghamii* | 21,33,55,77,99,127 | 79 | 1.54 | 47.02 | 12.88 |
| *Podocarpus macrophyllus* | 21,33,55,77,99,127 | 41 | 1.39 | 47.25 | 180.37 |
| *Ephedra przewalskii* | Illumina +nanopore | 15 | 0.45 | 46.61 | - |
| *Gnetum montanum* | 21,33,55,77,99,127 | 33 | 1.13 | 47.66 | 20.97 |
| *Welwitschia mirabilis* | 21,33,55,77,99,127 | 17 | 0.98 | 53.20 | 115.22 |
