# Supplementary material for: Both Conifer II and Gnetales are characterized by a high frequency of ancient mitochondrial gene transfer to the nuclear genome
Source: BMC Biol. 2021 Jul 28;19:146. doi: 10.1186/s12915-021-01096-z (PMC8317393; doi:10.1186/s12915-021-01096-z)
Supplement: Supplementary file 2 — Additional file 2: Table S2. Statistics of mitochondrial protein-coding genes in gymnosperms. [file 12915_2021_1096_MOESM2_ESM.docx]

**Additional file 2: Table S2.** Statistics of mitochondrial protein-coding genes in gymnosperms.

|  | ***Cyc*** | ***Zam*** | ***Gin*** | ***Pin*** | ***Pic*** | ***Abi*** | ***Ced*** | ***Eph*** | ***Gne*** | ***Wel*** | ***Pla*** | ***Met*** | ***Cun*** | ***Tai*** | ***Cep*** | ***Tax*** | ***Sci*** | ***Ara*** | ***Pod*** |
| --- | --- | --- | --- | --- | --- | --- | --- | --- | --- | --- | --- | --- | --- | --- | --- | --- | --- | --- | --- |
| *nad1* | • | • | • | • | • | • | • | • | • | • | • | • | • | • | • | • | • | • | • |
| *nad2* | • | • | • | • | • | • | • | • | • | • | • | • | • | • | • | • | • | • | • |
| *nad3* | • | • | • | • | • | • | • | • | • | • | • | • | • | • | • | • | • | • | • |
| *nad4* | • | • | • | • | • | • | • | • | • | • | • | • | • | • | • | • | • | • | • |
| *nad4L* | • | • | • | • | • | • | • | • | • | • | • | • | • | • | • | • | • | • | • |
| *nad5* | • | • | • | • | • | • | • | • | • | • | • | • | • | • | • | • | • | • | • |
| *nad6* | • | • | • | • | • | • | • | • | • | • | • | • | • | • | • | • | • | • | • |
| *nad7* | • | • | • | • | • | • | • | • | • | • | • | • | • | • | • | • | • | • | • |
| *nad9* | • | • | • | • | • | • | • | • | • | • | • | • | • | • | • | • | • | • | • |
| *cob* | • | • | • | • | • | • | • | • | • | • | • | • | • | • | • | • | • | • | • |
| *cox1* | • | • | • | • | • | • | • | • | • | • | • | • | • | • | • | • | • | • | • |
| *cox2* | • | • | • | • | • | • | • | • | • | • | • | • | • | • | • | • | • | • | • |
| *cox3* | • | • | • | • | • | • | • | • | • | • | • | • | • | • | • | • | • | • | • |
| *atp1* | • | • | • | • | • | • | • | • | • | • | • | • | • | • | • | • | • | • | • |
| *atp4* | • | • | • | • | • | • | • | • | • | • | • | • | • | • | • | • | • | • | • |
| *atp6* | • | • | • | • | • | • | • | • | • | • | • | • | • | • | • | • | • | • | • |
| *atp8* | • | • | • | • | • | • | • | • | • | • | • | • | • | • | • | • | • | • | • |
| *atp9* | • | • | • | • | • | • | • | • | • | • | • | • | • | • | • | • | • | • | • |
| *ccmB* | • | • | • | • | • | • | • | – | • | • | • | • | • | • | • | • | • | • | • |
| *ccmC* | • | • | • | • | • | • | • | • | • | • | • | • | • | • | • | • | • | • | • |
| *ccmFC* | • | • | • | • | • | • | • | • | • | • | • | • | • | • | • | • | • | • | • |
| *ccmFN* | • | • | • | • | • | • | • | • | • | • | • | • | • | • | • | • | • | • | • |
| *matR* | • | • | • | • | • | • | • | – | • | • | • | • | • | • | • | • | • | • | • |
| *mttB* | • | • | • | • | • | • | • | – | • | • | • | • | • | • | • | • | • | • | • |
| *sdh3* | • | • | • | • | • | • | • | t | t | t | t | t | t | t | t | t | t | • | • |
| *sdh4* | • | • | • | • | • | • | • | • | • | • | • | • | • | • | • | • | • | • | • |
| *rpl2* | • | • | • | • | • | • | • | – | – | – | t | t | t | t | t | t | t | t | t |
| *rpl5* | • | • | • | • | • | • | • | – | – | – | • | • | • | • | • | • | • | • | • |
| *rpl10* | • | • | • | • | • | • | • | – | • | • | – | – | – | – | – | – | • | – | – |
| *rpl16* | • | • | • | • | • | • | • | – | – | – | • | • | • | • | • | • | • | • | • |
| *rps1* | • | • | • | • | • | • | • | t | t | t | t | t | t | t | t | t | t | t | t |
| *rps2* | • | • | • | • | • | • | • | t | t | t | t | t | t | t | t | t | t | t | t |
| *rps3* | • | • | • | • | • | • | • | – | • | • | • | • | • | • | • | • | • | • | • |
| *rps4* | • | • | • | • | • | • | • | – | • | • | • | • | • | • | • | • | • | • | • |
| *rps7* | • | • | • | • | • | • | • | – | – | – | t | t | t | t | t | t | t | t | t |
| *rps10* | • | • | • | • | • | • | • | t | t | t | t | t | t | t | t | t | t | t | t |
| *rps11* | • | • | • | • | • | • | • | t | t | t | t | t | t | t | t | t | t | t | t |
| *rps12* | • | • | • | • | • | • | • | t | • | • | • | • | • | • | • | • | • | • | • |
| *rps13* | • | • | • | • | • | • | • | – | – | – | • | • | • | • | • | • | • | • | • |
| *rps14* | • | • | • | • | • | • | • | t | t | t | t | t | t | t | t | t | t | t | t |
| *rps19* | • | • | • | • | • | • | • | – | – | – | • | • | • | • | • | • | • | • | • |
| **Total (•)** | **41** | **41** | **41** | **41** | **41** | **41** | **41** | **22** | **29** | **29** | **32** | **32** | **32** | **32** | **32** | **32** | **33** | **33** | **33** |
| **Total (t)** | **0** | **0** | **0** | **0** | **0** | **0** | **0** | **7** | **6** | **6** | **8** | **8** | **8** | **8** | **8** | **8** | **8** | **7** | **7** |
| **Total (–)** | **0** | **0** | **0** | **0** | **0** | **0** | **0** | **12** | **6** | **6** | **1** | **1** | **1** | **1** | **1** | **1** | **0** | **1** | **1** |

(“•”indicates the presence of an intact gene in mitogenome; “t” indicates the presence of an intact gene in nuclear genome; “–” indicates gene loss)
