# Supplementary figures and images for: Both Conifer II and Gnetales are characterized by a high frequency of ancient mitochondrial gene transfer to the nuclear genome
Source: BMC Biol. 2021 Jul 28;19:146. doi: 10.1186/s12915-021-01096-z (PMC8317393; doi:10.1186/s12915-021-01096-z)

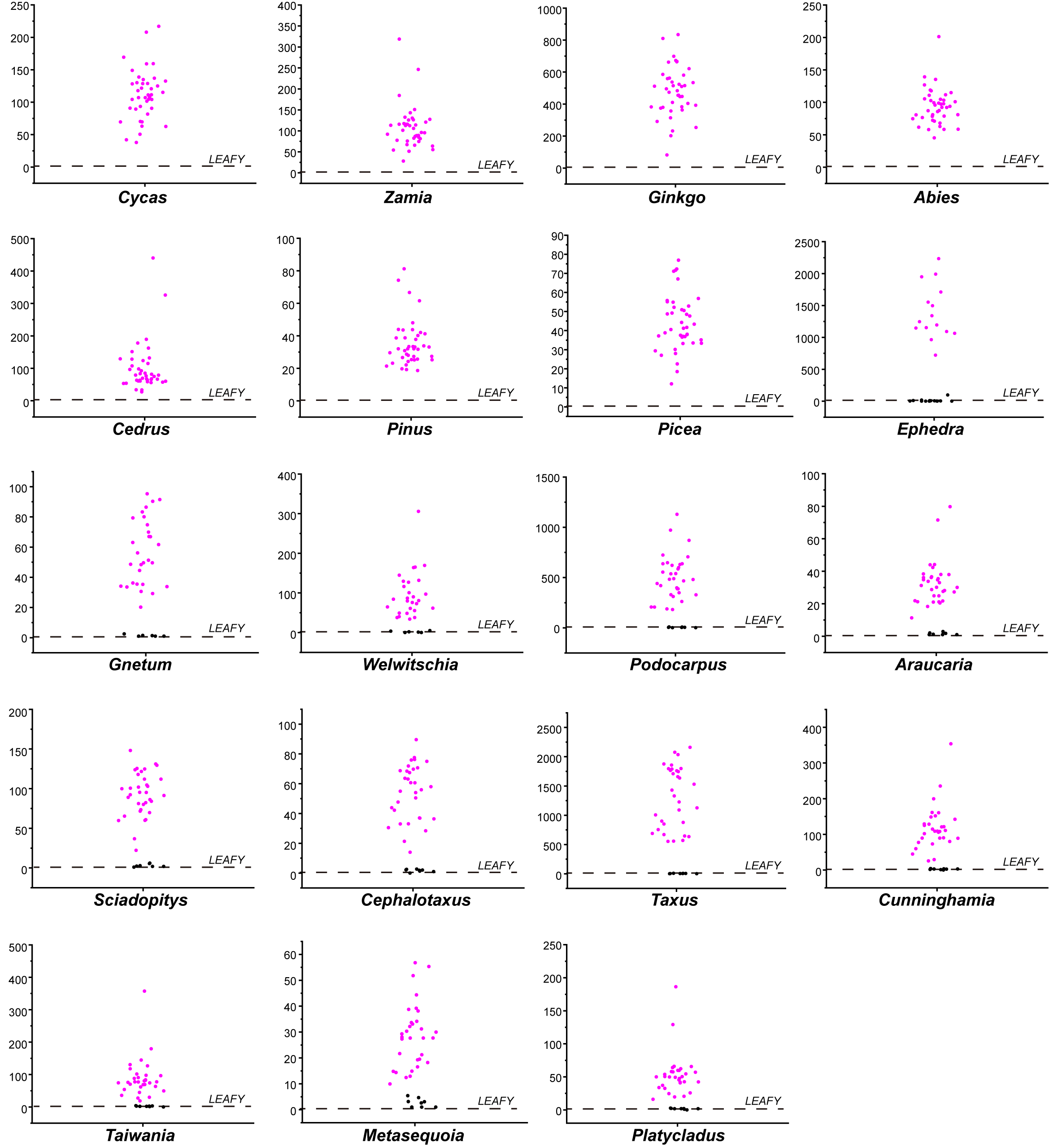

Supplement: Supplementary file 3 — Additional file 3: Figure S1. The sequencing depth of transferred and mitochondrial genes in gymnosperms. Purple and black indicate mitochondrial and transferred genes, respectively. [file 12915_2021_1096_MOESM3_ESM.tif]

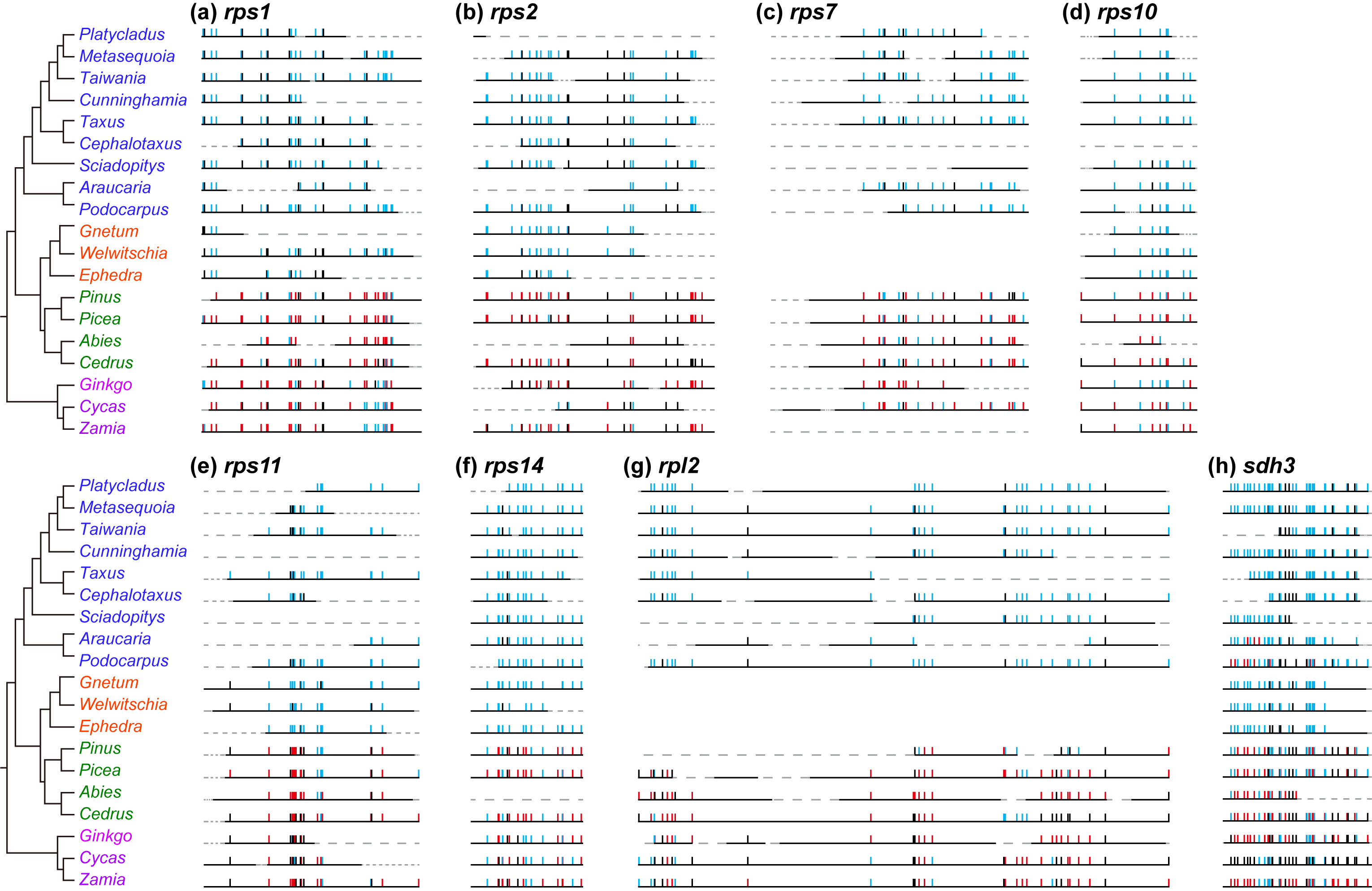

Supplement: Supplementary file 7 — Additional file 7: Figure S2. Localization of RNA editing sites of eight mitochondrial genes and the corresponding bases of their transferred homologs. The red vertical line indicates that the site was edited in the mitochondrial gene, the blue vertical line indicates that the site was converted from C to T in the DNA sequence, and the black vertical line indicates that the site was C in the DNA sequence and was not edited in the RNA sequence. a, rps1; b, rps2; c, rps7; d, rps10; e, rps11; f, rps14; g, rpl2; h, sdh3.) [file 12915_2021_1096_MOESM7_ESM.tif]

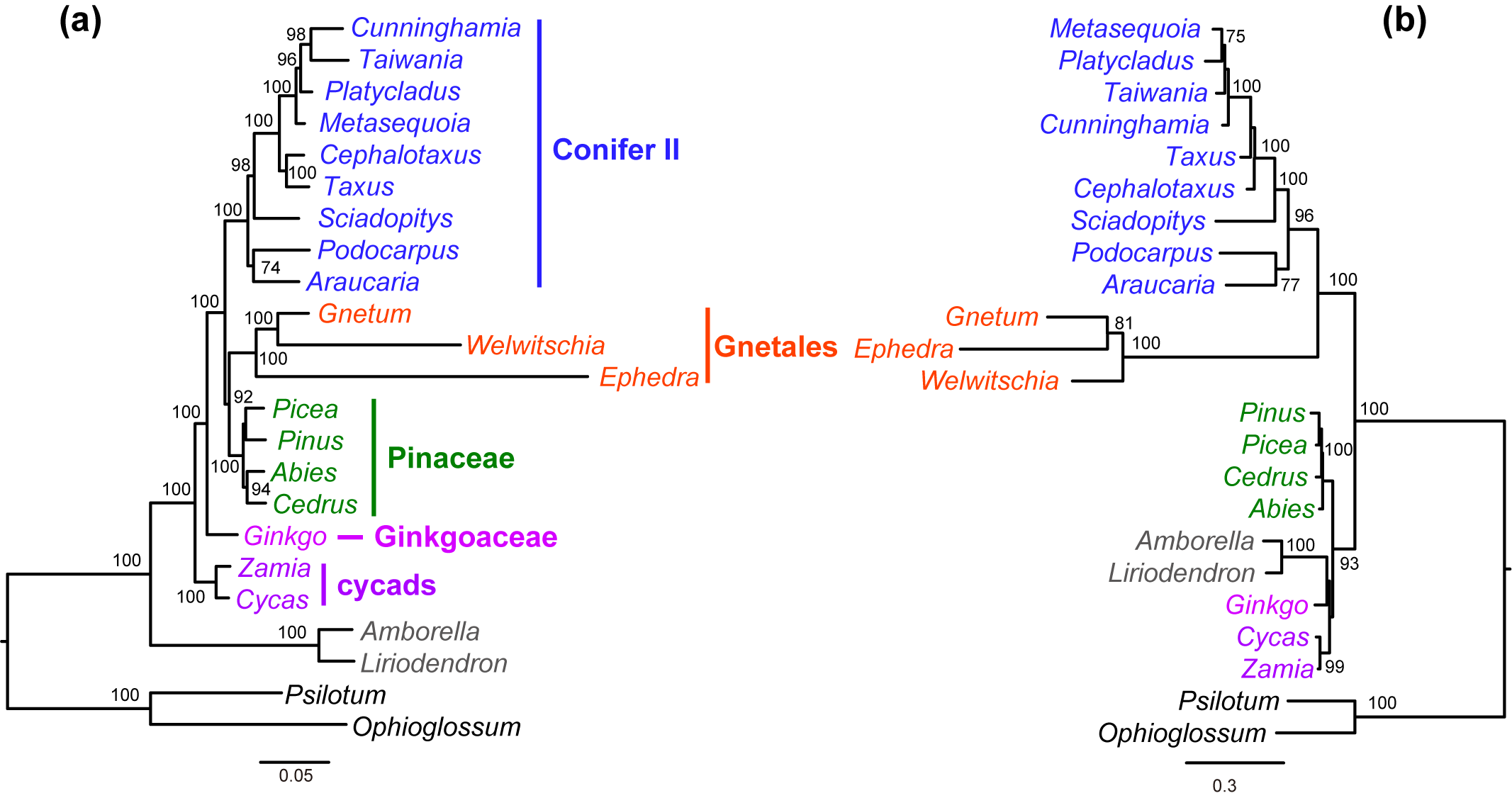

Supplement: Supplementary file 8 — Additional file 8: Figure S3. Phylogenetic relationships reconstructed by 22 mitochondrial genes (a) and 5 transferred genes and their mitochondrial homologs (b), respectively. [file 12915_2021_1096_MOESM8_ESM.tif]

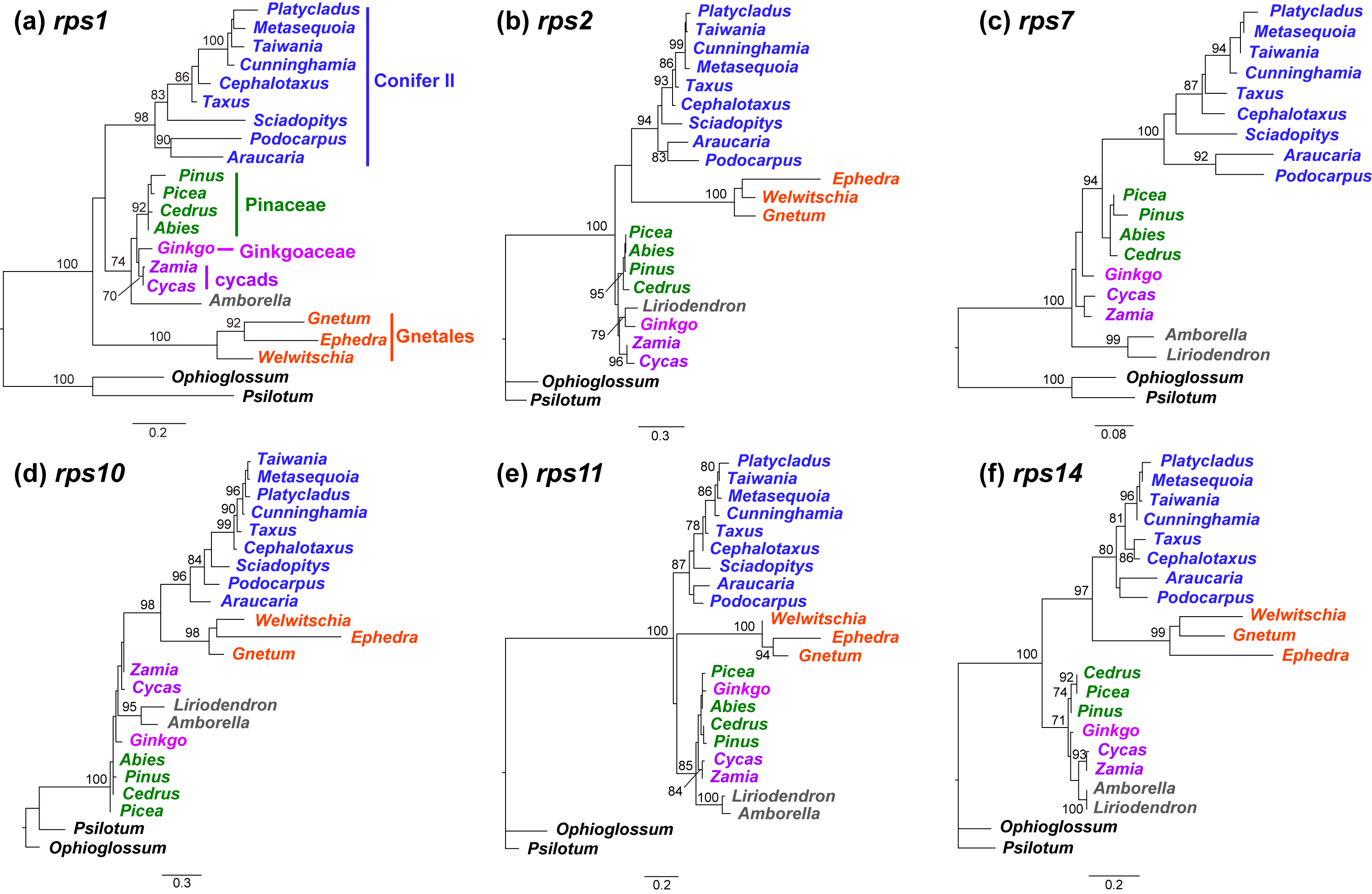

Supplement: Supplementary file 9 — Additional file 9: Figure S4. Single-gene tree reconstructed using transferred genes and their mitochondrial homologs. [file 12915_2021_1096_MOESM9_ESM.tif]

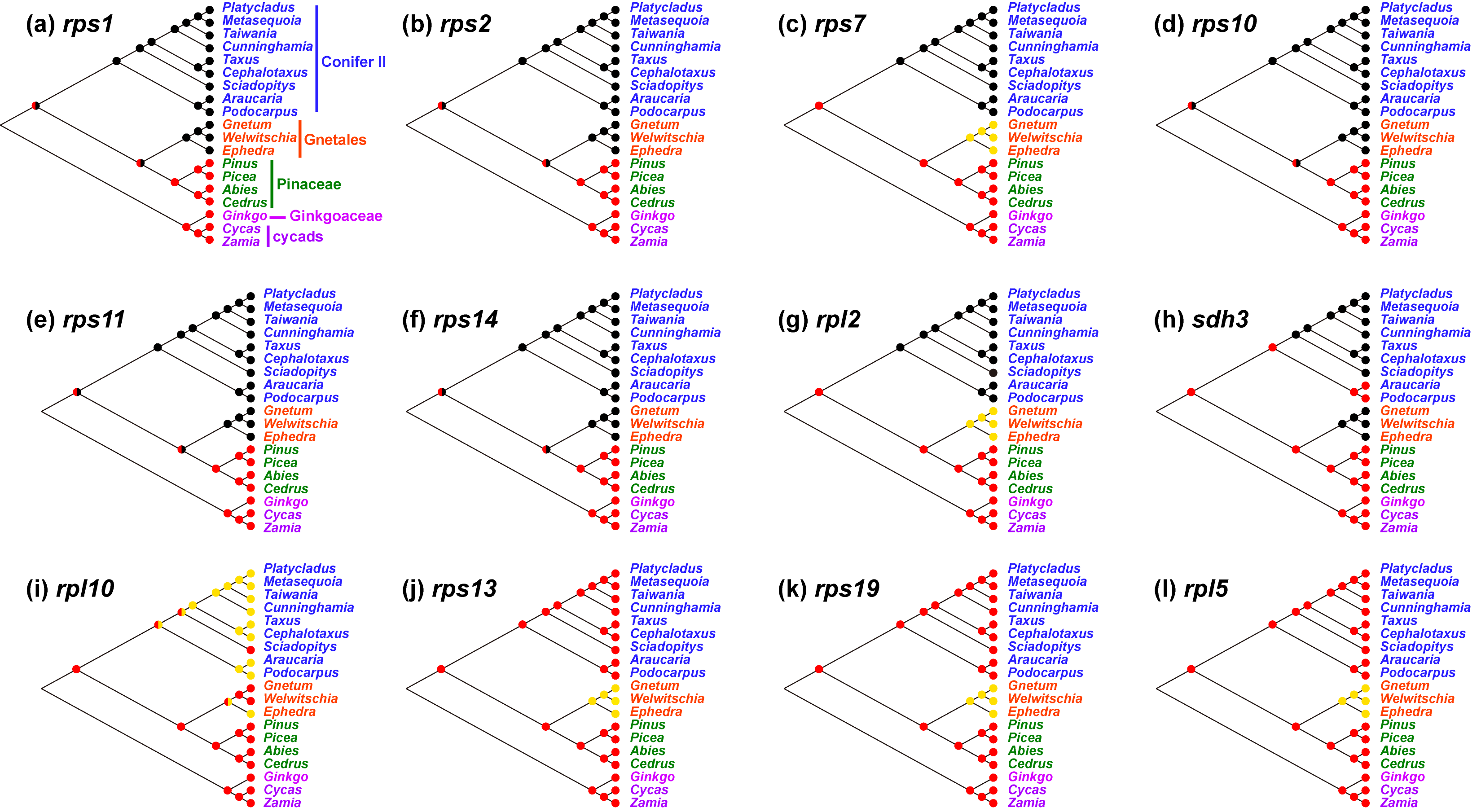

Supplement: Supplementary file 10 — Additional file 10: Figure S5. Ancestral state reconstruction of mitochondrial gene transfer/loss in gymnosperms. Yellow circle indicates lost gene, red circle indicates mitochondrial gene, and black circle indicates transferred gene. [file 12915_2021_1096_MOESM10_ESM.tif]

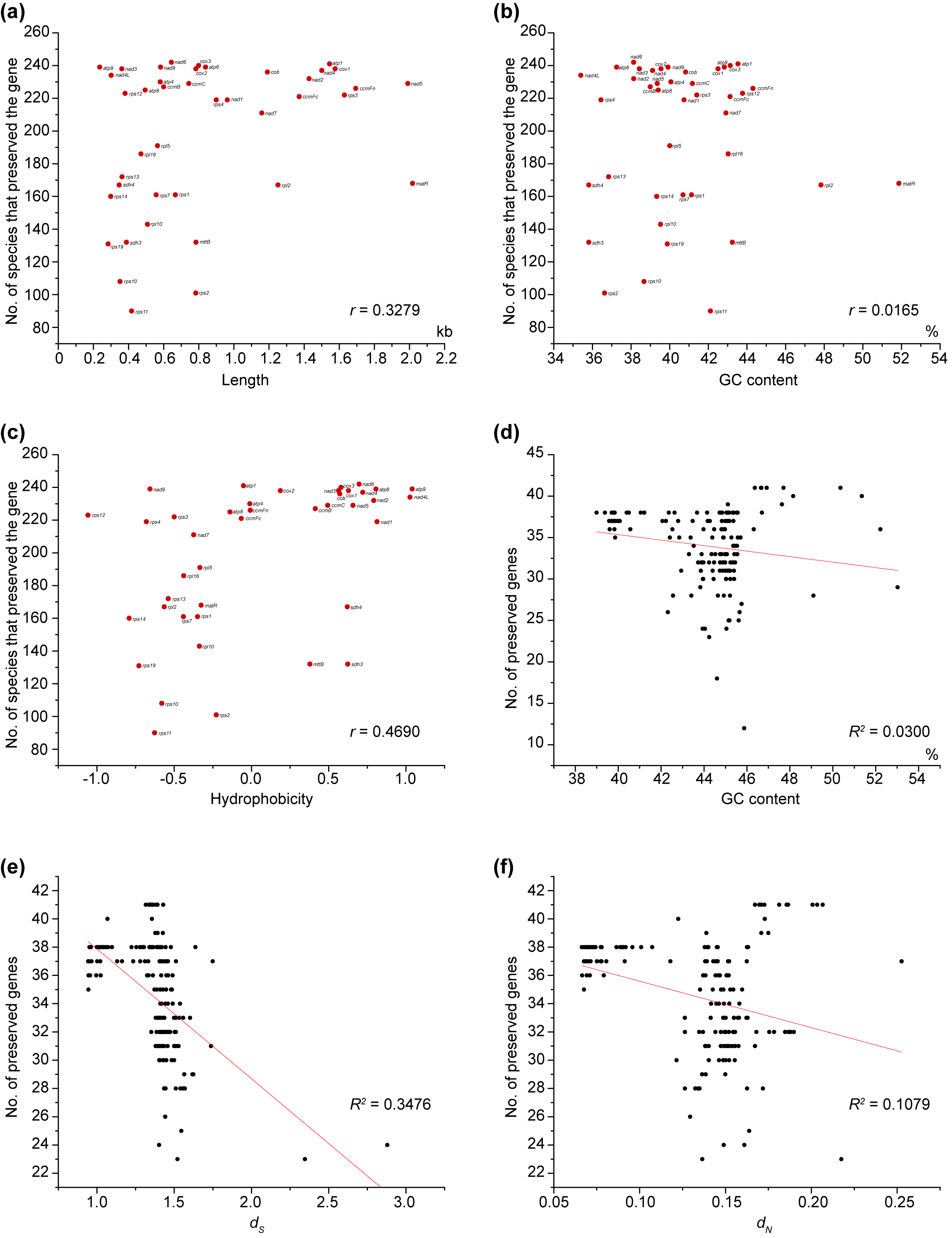

Supplement: Supplementary file 11 — Additional file 11: Figure S6. Correlation between the number of species that preserved the gene in land plants and gene length (a), GC content (b) and hydrophobicity (c) and correlation between number of mitochondrial genes in terrestrial plants and GC content (d) and substitution rate (e and f). r indicates the Pearson’s correlation coefficient, and R2 indicates the coefficient of determination in linear regression analysis. [file 12915_2021_1096_MOESM11_ESM.tif]
