# Supplementary material for: Both Conifer II and Gnetales are characterized by a high frequency of ancient mitochondrial gene transfer to the nuclear genome
Source: BMC Biol. 2021 Jul 28;19:146. doi: 10.1186/s12915-021-01096-z (PMC8317393; doi:10.1186/s12915-021-01096-z)
Supplement: Supplementary file 4 — Additional file 4: Table S3. Statistics of mitochondrial introns in gymnosperms. [file 12915_2021_1096_MOESM4_ESM.docx]

**Additional file 4: Table S3.** Statistics of mitochondrial introns in gymnosperms.

|  | ***Cyc*** | ***Zam*** | ***Gin*** | ***Pin*** | ***Pic*** | ***Abi*** | ***Ced*** | ***Eph*** | ***Gne*** | ***Wel*** | ***Pla*** | ***Met*** | ***Cun*** | ***Tai*** | ***Cep*** | ***Tax*** | ***Sci*** | ***Ara*** | ***Pod*** |
| --- | --- | --- | --- | --- | --- | --- | --- | --- | --- | --- | --- | --- | --- | --- | --- | --- | --- | --- | --- |
| ccmFci829 | • | • | • | • | • | • | • | – | • | – | – | – | – | – | – | – | – | – | – |
| cox2i373 | • | • | • | ɵ | ɵ | ɵ | ɵ | ɵ | – | – | ɵ | ɵ | ɵ | ɵ | ɵ | ɵ | ɵ | ɵ | ɵ |
| cox2i691 | • | • | • | ɵ | ɵ | ɵ | ɵ | – | ɵ | ɵ | – | – | – | – | – | – | – | – | – |
| nad1i394 | ɵ | ɵ | ɵ | ɵ | ɵ | ɵ | ɵ | ɵ | ɵ | ɵ | ɵ | ɵ | ɵ | ɵ | ɵ | ɵ | ɵ | ɵ | ɵ |
| nad1i477 | • | • | • | • | • | • | • | ɵ | • | – | – | – | – | – | – | – | – | – | – |
| nad1i669 | ɵ | ɵ | ɵ | ɵ | ɵ | ɵ | ɵ | ɵ | ɵ | ɵ | ɵ | ɵ | ɵ | ɵ | ɵ | ɵ | ɵ | ɵ | ɵ |
| nad1i728 | • | • | • | ɵ | ɵ | ɵ | ɵ | ɵ | ɵ | ɵ | ɵ | ɵ | ɵ | ɵ | ɵ | ɵ | ɵ | ɵ | ɵ |
| nad2i156 | • | • | • | • | • | • | • | ɵ | • | – | – | – | – | – | – | – | – | – | – |
| nad2i542 | ɵ | ɵ | ɵ | ɵ | ɵ | ɵ | ɵ | • | ɵ | ɵ | ɵ | ɵ | ɵ | ɵ | ɵ | ɵ | ɵ | ɵ | ɵ |
| nad2i709 | • | • | • | • | • | • | • | ɵ | • | – | • | • | • | • | ɵ | • | • | • | • |
| nad2i1282 | • | • | • | ɵ | ɵ | ɵ | ɵ | ɵ | • | – | ɵ | ɵ | ɵ | ɵ | ɵ | ɵ | ɵ | ɵ | ɵ |
| nad4i461 | • | • | • | ɵ | ɵ | ɵ | ɵ | ɵ | • | • | ɵ | ɵ | ɵ | ɵ | ɵ | ɵ | ɵ | ɵ | ɵ |
| nad4i976 | • | • | • | • | • | • | • | ɵ | • | – | ɵ | ɵ | ɵ | ɵ | ɵ | ɵ | ɵ | ɵ | ɵ |
| nad4i1399 | • | • | • | • | • | • | • | ɵ | • | – | ɵ | ɵ | ɵ | ɵ | ɵ | ɵ | ɵ | ɵ | ɵ |
| nad5i230 | • | • | • | • | • | • | • | ɵ | • | – | • | • | • | • | • | • | • | • | • |
| nad5i1455 | ɵ | ɵ | ɵ | ɵ | ɵ | ɵ | ɵ | ɵ | ɵ | ɵ | ɵ | ɵ | ɵ | ɵ | ɵ | ɵ | ɵ | ɵ | ɵ |
| nad5i1477 | ɵ | ɵ | ɵ | ɵ | ɵ | ɵ | ɵ | ? | ɵ | ɵ | ɵ | ɵ | ɵ | ɵ | ɵ | ɵ | ɵ | ɵ | – |
| nad5i1872 | • | • | • | • | • | • | • | ? | • | – | • | • | • | • | • | • | • | • | • |
| nad7i140 | • | • | • | • | • | • | • | ɵ | – | – | – | – | – | – | – | – | – | – | – |
| nad7i209 | • | • | • | ɵ | ɵ | ɵ | ɵ | ɵ | • | • | • | • | • | • | • | • | • | • | • |
| nad7i676 | • | • | • | • | • | • | • | ɵ | • | – | – | – | – | – | – | – | – | – | – |
| nad7i917 | • | • | • | ɵ | ɵ | ɵ | ɵ | ɵ | • | • | – | – | – | – | – | – | – | – | – |
| rpl2i917 | • | • | • | ɵ | ɵ | ɵ | ɵ | x | x | x | x | x | x | x | x | x | x | x | x |
| rps3i74 | • | • | • | • | • | • | • | x | • | – | – | – | – | – | – | – | – | – | – |
| rps3i249 | • | • | • | • | • | • | • | x | • | – | – | – | – | – | – | – | – | – | – |
| rps10i235 | • | • | – | • | • | • | • | x | x | x | x | x | x | x | x | x | x | x | x |
| **Total (•)** | **21** | **21** | **20** | **13** | **13** | **13** | **13** | **1** | **15** | **3** | **4** | **4** | **4** | **4** | **3** | **4** | **4** | **4** | **4** |
| **Total (ɵ)** | **5** | **5** | **5** | **13** | **13** | **13** | **13** | **17** | **7** | **7** | **11** | **11** | **11** | **11** | **12** | **11** | **11** | **11** | **10** |
| **Total (–)** | **0** | **0** | **1** | **0** | **0** | **0** | **0** | **2** | **2** | **14** | **9** | **9** | **9** | **9** | **9** | **9** | **9** | **9** | **10** |
| **Total (x)** | **0** | **0** | **0** | **0** | **0** | **0** | **0** | **4** | **2** | **2** | **2** | **2** | **2** | **2** | **2** | **2** | **2** | **2** | **2** |

(“•” indicates *cis*-spliced intron; “ɵ” indicates *trans*-spliced intron; “–” indicates intron loss from an intact gene; “x” indicates intron loss due to gene loss; “?” indicates that it is uncertain whether the intron present or not.)
