# Supplementary material for: Both Conifer II and Gnetales are characterized by a high frequency of ancient mitochondrial gene transfer to the nuclear genome
Source: BMC Biol. 2021 Jul 28;19:146. doi: 10.1186/s12915-021-01096-z (PMC8317393; doi:10.1186/s12915-021-01096-z)
Supplement: Supplementary file 5 — Additional file 5: Table S4. Annotation of the N-terminal presequences of putative transferred genes in gymnosperms. [file 12915_2021_1096_MOESM5_ESM.docx]

**Additional file 5: Table S4. Annotation of the N-terminal presequences of putative transferred genes in gymnosperms.**

| **gene** | **Species** | **Description** |
| --- | --- | --- |
| *rps1* | *Platycladus orientalis* | *Glycine max* probable LRR receptor-like serine/threonine-protein kinase At1g56130 (LOC100812068), mRNA |
|  | *Metasequoia glyptostroboides* | *Cryptomeria japonica* mRNA, clone: CMFL011_M16, expressed in male strobili |
|  | *Taiwania cryptomerioides* | *Cryptomeria japonica* mRNA, clone: CMFL011_M16, expressed in male strobili |
|  | *Cunninghamia lanceolata* | *Cryptomeria japonica* mRNA, clone: CMFL011_M16, expressed in male strobili |
|  | *Taxus cuspidata* | *Lupinus angustifolius* cultivar *Tanjil* chromosome LG-13 |
|  | *Cephalotaxus sinensis* | *Lupinus angustifolius* cultivar *Tanjil* chromosome LG-16 |
|  | *Sciadopitys verticillata* | - |
|  | *Araucaria cunninghamii* | *Arabidopsis lyrata* subsp. *lyrata* uncharacterized LOC9311815 (LOC9311815), transcript variant X2, mRNA |
|  | *Podocarpus macrophyllus* | *Caenorhabditis* sp. 34 TK-2017 DNA. chromosome 4, nearly complete genome |
|  | *Gnetum montanum* | *Solanum lycopersicum* cultivar I-3 chromosome 2 |
|  | *Welwitschia mirabilis* | PREDICTED: *Olea europaea* var. *sylvestris* uncharacterized LOC111377756 (LOC111377756), ncRNA |
|  | *Ephedra przewalskii* | *Nelumbo nucifera* pentatricopeptide repeat-containing protein At5g50390, chloroplastic (LOC104590160), transcript variant X2, mRNA |
| *rps2* | *Platycladus orientalis* | *Medicago truncatula* clone mth2-43p1, complete sequence |
|  | *Metasequoia glyptostroboides* | *Cryptomeria japonica* mRNA, clone: CLFL024_I02, expressed in needles |
|  | *Taiwania cryptomerioides* | *Cryptomeria japonica* mRNA, clone: CFFL048_E13, expressed in female strobili |
|  | *Cunninghamia lanceolata* | *Gossypium hirsutum* cultivar *TM1* chromosome D11 |
|  | *Taxus cuspidata* | *Impatiens glandulifera* genome assembly, chromosome: 7 |
|  | *Cephalotaxus sinensis* | *Triticum aestivum* chromosome 3B, genomic scaffold, cultivar Chinese Spring |
|  | *Sciadopitys verticillata* | *Lupinus angustifolius* cultivar *Tanjil* chromosome LG-20 |
|  | *Araucaria cunninghamii* | *Solanum pennellii* chromosome ch09, complete genome |
|  | *Podocarpus macrophyllus* | *Lupinus angustifolius* cultivar *Tanjil* chromosome LG-13 |
|  | *Gnetum montanum* | *Gossypium turneri* isolate D10-2 chromosome D10_11 |
|  | *Welwitschia mirabilis* | PREDICTED: *Camelina sativa* translation initiation factor eIF-2B subunit epsilon-like (LOC104786056), mRNA |
|  | *Ephedra przewalskii* | *Helianthus annuus* cysteine-rich repeat secretory protein 12-like (LOC110894404), mRNA |
| *rps7* | *Platycladus orientalis* | *Vitis vinifera* contig VV78X078957.12, whole genome shotgun sequence |
|  | *Metasequoia glyptostroboides* | - |
|  | *Taiwania cryptomerioides* | - |
|  | *Cunninghamia lanceolata* | *Cryptomeria japonica* mRNA, clone: CLFL047_D10, expressed in needles |
|  | *Taxus cuspidata* | *Impatiens glandulifera* genome assembly, chromosome: 9 |
|  | *Cephalotaxus sinensis* | *Dracunculus medinensis* genome assembly D_medinensis_Ghana, scaffold DME_scaffold0000021 |
|  | *Sciadopitys verticillata* | - |
|  | *Araucaria cunninghamii* | *Cucurbita pepo* subsp. *pepo* uncharacterized LOC111796927 (LOC111796927), mRNA |
|  | *Podocarpus macrophyllus* | - |
| *rps10* | *Platycladus orientalis* | *Cryptomeria japonica* mRNA, clone: CFFL042_K11, expressed in female strobili |
|  | *Metasequoia glyptostroboides* | *Cryptomeria japonica* mRNA, clone: CFFL042_K11, expressed in female strobili |
|  | *Taiwania cryptomerioides* | *Cryptomeria japonica* mRNA, clone: CFFL027_D02, expressed in female strobili |
|  | *Cunninghamia lanceolata* | *Cryptomeria japonica* mRNA, clone: CFFL042_K11, expressed in female strobili |
|  | *Taxus cuspidata* | PREDICTED: *Quercus suber* putative pentatricopeptide repeat-containing protein At1g12700, mitochondrial (LOC112025772), transcript variant X3, mRNA |
|  | *Cephalotaxus sinensis* | PREDICTED: *Brassica napus* bZIP transcription factor 44-like (LOC106353536), mRNA |
|  | *Sciadopitys verticillata* | *Oryza sativa* Indica Group cultivar *Shuhui498* chromosome 4 sequence |
|  | *Araucaria cunninghamii* | - |
|  | *Podocarpus macrophyllus* | - |
|  | *Gnetum montanum* | *Solanum lycopersicum* cultivar *I-3* chromosome 5 |
|  | *Welwitschia mirabilis* | *Impatiens glandulifera* genome assembly, chromosome: 8 |
|  | *Ephedra przewalskii* | *Sorghum bicolor* THO complex subunit 1 (LOC8078017), transcript variant X3, mRNA |
| *rps11* | *Platycladus orientalis* | - |
|  | *Metasequoia glyptostroboides* | PREDICTED: *Amborella trichopoda* histone H3.2 (LOC110006361), mRNA |
|  | *Taiwania cryptomerioides* | *Medicago truncatula* chromosome 5 clone mth4-42c9, COMPLETE SEQUENCE |
|  | *Cunninghamia lanceolata* | *Solanum lycopersicum* cultivar *I-3* chromosome 5 |
|  | *Taxus cuspidata* | *Taxus* x media 5-alpha-taxadienol-10-beta-hydroxylase (10bh) mRNA, complete cds |
|  | *Cephalotaxus sinensis* | - |
|  | *Sciadopitys verticillata* | - |
|  | *Araucaria cunninghamii* | *Herrania umbratica* guanylate-binding protein 5 (LOC110414372), mRNA |
|  | *Podocarpus macrophyllus* | *Solanum lycopersicum* cultivar *I-3* chromosome 5 |
|  | *Gnetum montanum* | *Solanum lycopersicum* cultivar *I-3* chromosome 4 |
|  | *Welwitschia mirabilis* | *Picea glauca* clone GQ02804_M05 mRNA sequence |
|  | *Ephedra przewalskii* | *Picea glauca* clone GQ02510_E13 mRNA sequence |
| *rps14* | *Platycladus orientalis* | *Cryptomeria japonica* mRNA, clone: CLFL005_P12, expressed in needles |
|  | *Metasequoia glyptostroboides* | *Cryptomeria japonica* mRNA, clone: CLFL005_P12, expressed in needles |
|  | *Taiwania cryptomerioides* | *Cryptomeria japonica* mRNA, clone: CLFL005_P12, expressed in needles |
|  | *Cunninghamia lanceolata* | *Cryptomeria japonica* mRNA, clone: CLFL005_P12, expressed in needles |
|  | *Taxus cuspidata* | *Vitis vinifera* contig VV78X170716.10, whole genome shotgun sequence |
|  | *Cephalotaxus sinensis* | *Cryptomeria japonica* mRNA, clone: CLFL005_P12, expressed in needles |
|  | *Sciadopitys verticillata* | *Picea glauca* clone GQ03312_F04 mRNA sequence |
|  | *Araucaria cunninghamii* | - |
|  | *Podocarpus macrophyllus* | PREDICTED: *Zea mays* ABC transporter C family member 8 (LOC103638451), mRNA |
|  | *Gnetum montanum* | *Marchantia polymorpha* subsp. *ruderalis* Tak-1 DNA, chromosome: 4 |
|  | *Welwitschia mirabilis* | *Vitis vinifera* contig VV78X187971.25, whole genome shotgun sequence |
|  | *Ephedra przewalskii* | *Cucumis melo* genomic chromosome, chr_3 |
| *rpl2* | *Platycladus orientalis* | *Cryptomeria japonica* mRNA, clone: CSFL014_K19, expressed in reproductive shoots |
|  | *Metasequoia glyptostroboides* | *Cryptomeria japonica* mRNA, clone: CSFL014_K19, expressed in reproductive shoots |
|  | *Taiwania cryptomerioides* | *Cryptomeria japonica* mRNA, clone: CSFL014_K19, expressed in reproductive shoots |
|  | *Cunninghamia lanceolata* | *Cryptomeria japonica* mRNA, clone: CSFL014_K19, expressed in reproductive shoots |
|  | *Taxus cuspidata* | *Picea glauca* clone GQ04009_G13 mRNA sequence |
|  | *Cephalotaxus sinensis* | *Solanum pennellii* chromosome ch11, complete genome |
|  | *Sciadopitys verticillata* | *Vitis vinifera* contig VV78X173468.5, whole genome shotgun sequence |
|  | *Araucaria cunninghamii* | - |
|  | *Podocarpus macrophyllus* | *Vitis vinifera* contig VV78X120913.3, whole genome shotgun sequence |
| *sdh3* | *Platycladus orientalis* | PREDICTED: *Asparagus officinalis* heat shock 70 kDa protein, mitochondrial (LOC109851228), mRNA |
|  | *Metasequoia glyptostroboides* | PREDICTED: *Ipomoea nil* heat shock 70 kDa protein, mitochondrial (LOC109165234), mRNA |
|  | *Taiwania cryptomerioides* | PREDICTED: *Nicotiana tomentosiformis* heat shock 70 kDa protein, mitochondrial-like (LOC104103161), mRNA |
|  | *Cunninghamia lanceolata* | PREDICTED: *Nicotiana tomentosiformis* heat shock 70 kDa protein, mitochondrial-like (LOC104103161), mRNA |
|  | *Taxus cuspidata* | *Picea glauca* clone GQ04013_L12 mRNA sequence |
|  | *Cephalotaxus sinensis* | PREDICTED: *Dendrobium catenatum* heat shock 70 kDa protein, mitochondrial-like (LOC110110271), mRNA |
|  | *Sciadopitys verticillata* | - |
|  | *Gnetum montanum* | *Solanum pennellii* chromosome ch10, complete genome |
|  | *Welwitschia mirabilis* | *Picea sitchensis* clone WS0288_O11 unknown mRNA |
|  | *Ephedra przewalskii* | - |

-: The N-terminal presequence was not retrieved.
