# Supplementary material for: Both Conifer II and Gnetales are characterized by a high frequency of ancient mitochondrial gene transfer to the nuclear genome
Source: BMC Biol. 2021 Jul 28;19:146. doi: 10.1186/s12915-021-01096-z (PMC8317393; doi:10.1186/s12915-021-01096-z)
Supplement: Supplementary file 6 — Additional file 6: Table S5. Statistics of RNA editing sites in mitochondrial genome of gymnosperms. [file 12915_2021_1096_MOESM6_ESM.docx]

**Additional file 6: Table S5.** Statistics of RNA editing sites in mitochondrial genome of gymnosperms.

|  | RNA editing sites | Alignment length | Rate |
| --- | --- | --- | --- |
| *Platycladus orientalis* | 458 | 14597 | 3.14% |
| *Metasequoia glyptostroboides* | 470 | 12090 | 3.89% |
| *Taiwania cryptomerioides* | 159 | 4832 | 3.29% |
| *Cunninghamia lanceolata* | 140 | 4292 | 3.26% |
| *Taxus cuspidata* | 196 | 5625 | 3.48% |
| *Cephalotaxus sinensis* | 368 | 11477 | 3.21% |
| *Sciadopitys verticillata* | 70 | 4971 | 1.41% |
| *Araucaria cunninghamii* | 264 | 12890 | 2.05% |
| *Podocarpus macrophyllus* | 326 | 12556 | 2.60% |
| *Gnetum montanum* | 130 | 8243 | 1.58% |
| *Welwitschia mirabilis* | 0 | 1361 | 0.00% |
| *Ephedra przewalskii* | 25 | 1173 | 2.13% |
| *Abies firma* | 284 | 6861 | 4.14% |
| *Cedrus deodara* | 720 | 24058 | 2.99% |
| *Pinus armandii* | 458 | 12717 | 3.60% |
| *Picea smithiana* | 537 | 14658 | 3.66% |
| *Ginkgo biloba* | 315 | 12744 | 2.47% |
| *Cycas revoluta* | 429 | 12535 | 3.42% |
| *Zamia furfuracea* | 836 | 21967 | 3.81% |
