# Supplementary material for: Both Conifer II and Gnetales are characterized by a high frequency of ancient mitochondrial gene transfer to the nuclear genome
Source: BMC Biol. 2021 Jul 28;19:146. doi: 10.1186/s12915-021-01096-z (PMC8317393; doi:10.1186/s12915-021-01096-z)
Supplement: Supplementary file 12 — Additional file 12: Table S6. Samples and data used in this study. [file 12915_2021_1096_MOESM12_ESM.docx]

**Additional file 12: Table S6. Samples and data used in this study**

| **Family** | **Species** | **Abbr.** | **Vouchers** | **DNA-Seq** | | | | | **RNA-Seq** | |
| --- | --- | --- | --- | --- | --- | --- | --- | --- | --- | --- |
|  |  |  |  | **SRA number** | **Insert size** | **No of**  **raw reads** | **No of reads after filtering** | **Sources** | **SRA number** | **Sources** |
| Cycadaceae | *Cycas revoluta* | Cyc | IBCAS | SRR12710844 | 500~600bp | 105,498,850 | 92,365,440 | this study | SRX3059933 | Ran et al. 2018a |
| Zamiaceae | *Zamia furfuracea* | Zam | IBCAS | SRR12710843 | 500~600bp | 321,277,828 | 255,737,946 | this study | SRX3059934 | Ran et al. 2018a |
| Ginkgoaceae | *Ginkgo biloba* | Gin | IBCAS | SRR12710832 | 500~600bp | 244,202,532 | 190,363,586 | this study | SRX3059931 | Ran et al. 2018a |
| Pinaceae | *Pinus armandii* | Pin | IBCAS | SRR12710830 | 500~600bp | 230,699,476 | 205,041,406 | this study | SRX3057314 | Ran et al. 2018a |
|  | *Picea smithiana* | Pic | IBCAS | SRR12710829 | 500~600bp | 331,790,848 | 253,894,426 | this study | SRX3052146 | Ran et al. 2018a |
|  | *Abies firma* | Abi | IBCAS | SRR12710828 | 500~600bp | 118,708,292 | 102,318,956 | this study | SRX3055044 | Ran et al. 2018a |
|  | *Cedrus deodara* | Ced | IBCAS | SRR12710827 | 500~600bp | 146,022,268 | 125,392,740 | this study | SRX3744356 | Ran et al. 2018b |
| Cupressaceae | *Platycladus orientalis* | Pla | IBCAS | SRR12710826 | 500~600bp | 98,764,344 | 86,731,380 | this study | SRX3058062 | Ran et al. 2018a |
|  | *Metasequoia glyptostroboides* | Met | IBCAS | SRR12710825 | 500~600bp | 96,296,876 | 84,744,556 | this study | SRX3058056 | Ran et al. 2018a |
|  | *Cunninghamia lanceolata* | Cun | KIBCAS | SRR12710824 | 500~600bp | 121,189,994 | 102,091,770 | this study | SRX3058052 | Ran et al. 2018a |
|  | *Taiwania cryptomerioides* | Tai | KIBCAS | SRR12710842 | 500~600bp | 92,979,188 | 76,287,452 | this study | SRR12710841 | This study |
| Cephalotaxaceae | *Cephalotaxus sinensis* | Cep | IBCAS | SRR12710840 | 500~600bp | 123,759,340 | 110,057,302 | this study | SRX3057874 | Ran et al. 2018a |
| Taxaceae | *Taxus cuspidata* | Tax | IBCAS | SRR10305026 | 500~600bp | 225,648,634 | 161,440,724 | Kan et al. 2021 | SRR10305025 | Kan et al. 2021 |
|  |  |  |  | SRR10305024 | > 20kb | 2,642,193 | 2,296,952 | Kan et al. 2021 |  |  |
| Sciadopityaceae | *Sciadopitys verticillata* | Sci | KIBCAS | SRR12710839 | 500~600bp | 205,817,316 | 178,741,490 | this study | SRX3058164 | Ran et al. 2018a |
| Araucariaceae | *Araucaria cunninghamii* | Ara | IBCAS | SRR12710838 | 500~600bp | 129,595,388 | 118,215,136 | this study | SRX3057826 | Ran et al. 2018a |
| Podocarpaceae | *Podocarpus macrophyllus* | Pod | IBCAS | SRR12710837 | 500~600bp | 154,076,138 | 79,861,628 | this study | SRX3058149 | Ran et al. 2018a |
| Ephedraceae | *Ephedra* *przewalskii* | Eph | IBCAS | SRR12710836 | 500~600bp | 124,079,300 | 105,476,056 | this study | SRR12710834 | this study |
|  |  |  |  | SRR12710835 | > 20kb | 1,552,048 | 1,369,829 | this study |  |  |
| Gnetaceae | *Gnetum montanum* | Gne | Hainan, China | SRR12710833 | 500~600bp | 46,949,704 | 40,658,178 | this study | SRX3059920 | Ran et al. 2018a |
| Welwitschiaceae | *Welwitschia mirabilis* | Wel | Buy from Internet | SRR12710831 | 500~600bp | 74,248,360 | 45,301,194 | this study | SRX3059925 | Ran et al. 2018a |

IBCAS: Institute of Botany, the Chinese Academy of Sciences, Beijing, China; KIBCAS: Kunming Institute of Botany, the Chinese Academy of Sciences, Yunnan, China

Kan SL, Shen TT, Gong P, Ran JH, Wang XQ. The complete mitochondrial genome of *Taxus cuspidata* (Taxaceae): eight protein-coding genes have transferred to the nuclear genome. BMC Evol Biol. 2020;20:10.

Ran JH, Shen TT, Wang MM, Wang XQ. Phylogenomics resolves the deep phylogeny of seed plants and indicates partial convergent or homoplastic evolution between Gnetales and angiosperms. Proc R Soc B. 2018;285(1881):20181012.

Ran JH, Shen TT, Wu H, Gong X, Wang XQ. Phylogeny and evolutionary history of Pinaceae updated by transcriptomic analysis. Mol Phylogenet Evol. 2018;129:106-116.
