# Supplementary material for: Both Conifer II and Gnetales are characterized by a high frequency of ancient mitochondrial gene transfer to the nuclear genome
Source: BMC Biol. 2021 Jul 28;19:146. doi: 10.1186/s12915-021-01096-z (PMC8317393; doi:10.1186/s12915-021-01096-z)
Supplement: Supplementary file 13 — Additional file 13: Table S7. Primers used in this study. [file 12915_2021_1096_MOESM13_ESM.docx]

**Additional file 13: Table S7. Primers used in this study**

| **Species** | **Gene** | **Primer** | **Sequence (5' to 3')** | **Amplification efficiency (%)** |
| --- | --- | --- | --- | --- |
| **real-time PCR** |  |  |  |  |
| *Pinus armandii* | *LFY* | LFYE1F4 | CTGAACAAGAGCTGGATGATCTAGT | 98 |
|  |  | LFYE1R1 | GCATTCTCATCAATCTTGCGT |  |
|  | *atp1* | atp1_Pin_qF1 | AGCTGCGGAACTCACTACTCTA | 89 |
|  |  | atp1_Pin_qR1 | CTACCAAATACCACGATACCTACA |  |
|  | *atp4* | atp4_Pin_qF1 | CTCGTCGTTCCATAGGCTTTC | 91 |
|  |  | atp4_Pin_qR1 | CTGACTCCACATTTAGGTTTCG |  |
|  | *atp6* | atp6_Pin_qF1 | TTGAGCAATCTGCCATTATCCC | 89 |
|  |  | atp6_Pin_qR1 | GACCACCTATTTGTTCGTTCACC |  |
|  | *atp8* | atp8_Pin_qF1 | TAAGTGGCTCACGGATAATGGA | 90 |
|  |  | atp8_Pin_qR2 | AACGATGTTCATTTGGCCGTGT |  |
|  | *atp9* | atp9_Pin_qF1 | GAAACGCTCTTAGTTCCTTGAT | 94 |
|  |  | atp9_Pin_qR1 | AATGAGATCAGAAACGCCATC |  |
|  | *ccmB* | ccmB_Pin_qF1 | CGAACCTTTCTCTCGGAGTGAT | 94 |
|  |  | ccmB_Pin_qR1 | AGCCGAACGAGAATGAATACC |  |
|  | *ccmC* | ccmC_Pin_qF1 | CTTCCTGTCGAACCGGCTCCTA | 99 |
|  |  | ccmC_Pin_qR1 | TACCAGATTGGCTAATGCTCCC |  |
|  | *ccmFC* | ccmFC_Pin_qF1 | TTCAAGAAACGACGAGCACAAC | 91 |
|  |  | ccmFC_Pin_qR1 | GGAGCCAACAGAAATCCTATCC |  |
|  | *ccmFN* | ccmFN_Pin_qF2 | AGCACTGCACTTGGCACGAGA | 94 |
|  |  | ccmFN_Pin_qR2 | AGCGGTTCAGTACGAACGAATG |  |
|  | *cob* | cob_Pin_qF1 | TCCGCTATCTTCCCTTCCATTT | 88 |
|  |  | cob_Pin_qR1 | TCCGCCAAGAACAACCGAAATA |  |
|  | *cox1* | cox1_Pin_qF1 | CAGCCTCGGTAGAAGTGGGTAG | 91 |
|  |  | cox1_Pin_qR1 | CCGCGCATGTTGGGGATAGTAG |  |
|  | *cox2* | cox2_Pin_qF1 | GAAGAAATCCAATCCCGCAAAG | 91 |
|  |  | cox2_Pin_qR1 | ACCTCGTCCATCGAGTATAACG |  |
|  | *cox3* | cox3_Pin_qF2 | ATCGTATCGGAGGTCATGTTCT | 90 |
|  |  | cox3_Pin_qR1 | TAAACTGCTTGTTGCTCTTTCC |  |
|  | *mttB* | mttB_Pin_qF2 | TACGGAGAACGAAGGGCGAGAT | 90 |
|  |  | mttB_Pin_qR2 | TACCCGCAAAGCATGGGAAGTG |  |
|  | *matR* | matR_Pin_qF1 | ATCCCAAGTTCTTTCGCCTCAC | 92 |
|  |  | matR_Pin_qR1 | TCGGAATTTCGTGCTTCTGTCG |  |
|  | *nad1* | nad1_Pod_qE1F1 | GTAGCCTTCTTAGTGTTAGCCGA | 91 |
|  |  | nad1_Pin_qE1R1 | CCCGAGCAACCAAACTTAGCAT |  |
|  | *nad2* | nad2_Tax_qE5F1 | CTTAGCGAAAATAATGTATCCCG | 93 |
|  |  | nad2_Tax_qE5R1 | TGATGAGTAACTAAGAACGAGGGAG |  |
|  | *nad3* | nad3_Pin_qF1 | TCAGTTCGCTAGTTTCTTTGATCCC | 92 |
|  |  | nad3_Pod_qR1 | CGAAACGACTTCTGGCATCACC |  |
|  | *nad4* | nad4_Pod_qE2F1 | GATCAGGGCAGCATATCAGTTT | 90 |
|  |  | nad4_Pin_qE2R1 | CTTCGGGTAACCAGATATGAACT |  |
|  | *nad4L* | nad4L_Pin_qF1 | CTTTCGGTATTTCCCGTTTATC | 92 |
|  |  | nad4L_Pod_qR1 | ATGCGATTGATAGATTCTACTGC |  |
|  | *nad5* | nad5_Pin_qF1 | GGGCTTGCTTCCTCGTTACCTT | 88 |
|  |  | nad5_Pin_qR1 | CGGAAAGAGTGATAAGGAGTGGAA |  |
|  | *nad6* | nad6_Pin_qF1 | ACATCGGGCAATTCACTTCATAC | 93 |
|  |  | nad6_Pod_qR1 | TTGGTCTGTCGTCCTCCTCATT |  |
|  | *nad7* | nad7_Pin_qE5F1 | ATACCGCAGTTGAAGCACCTAA | 90 |
|  |  | nad7_Pin_qE5R1 | GAGTCCTTGTAAATGGGCAGAG |  |
|  | *nad9* | nad9_Pin_qF1 | CCGATACGGACTACCCATTTCA | 91 |
|  |  | nad9_Pin_qR1 | TCCCATACTTCTCGCTCCCACC |  |
|  | *rpl2* | rpl2_Pin_qF1 | AGCAGGCAAGATGGTGACGAAT | 95 |
|  |  | rpl2_Pin_qR1 | TGCTGTGCGAACAAGGAAAGGT |  |
|  | *rpl5* | rpl5_Pin_qF1 | AGTAGTTCCAAAGGCACTCCCA | 90 |
|  |  | rpl5_Pin_qR1 | GACATATCCCGTGTCTTTCTCG |  |
|  | *rpl16* | rpl16_Pin_qF1 | AAACCTACAGAAGTGAGAATGG | 92 |
|  |  | rpl16_Pin_qR1 | TCGAGCATCTGACAAACTAAC |  |
|  | *rps1* | rps1_Pin_qF1 | AATAGGAGGGTGAAAGGGTCTA | 94 |
|  |  | rps1_Pin_qR1 | GGGTTAATGTTCTCAATGGTGG |  |
|  | *rps2* | rps2_Pin_qF1 | AGATAGGGAGTCCTCGGTCATA | 92 |
|  |  | rps2_Pin_qR1 | AACTGTATAGAATCATTCGCTGGA |  |
|  | *rps3* | rps3_Pin_qE3F1 | GTGGGATAGGAGGATTGATGAG | 88 |
|  |  | rps3_Pin_qE3R1 | CGACCCGATTAAGGTATTAGTGT |  |
|  | *rps4* | rps4_Pin_qF1 | CCGTGAAACTATTCCTCAAGCG | 93 |
|  |  | rps4_Pin_qR1 | CTCCAGTAGGCGGAACCATCT |  |
|  | *rps7* | rps7_Pin_qF1 | TGCGAAGTGGGGAAAGTACGAG | 90 |
|  |  | rps7_Pin_qR2 | ACGTCGTTTGAAAGCTGCTCCA |  |
|  | *rps10* | rps10_Pin_qF1 | CCGCCAAGATACGCATAGTGA | 93 |
|  |  | rps10_Pin_qR1 | ACTCGTTCTGAAGGCAATCCAA |  |
|  | *rps11* | rps11_Pin_qF1 | CCCACCAATCCACAATAATACC | 92 |
|  |  | rps11_Pin_qR1 | TCACTACAACTGGCTTCCCTC |  |
|  | *rps12* | rps12_Pin_qF1 | TCGGAGAAATGTCCTCAGAAGC | 90 |
|  |  | rps12_Pod_qR1 | TGTTCCTGCGGATTATGACCTT |  |
|  | *rps13* | rps13_Pin_qF1 | AAGAAGCCACTCAGGTTCGTTA | 92 |
|  |  | rps13_Pin_qR1 | GCGACAAGTCCTAGCATTAGTATG |  |
|  | *rps14* | rps14_Pin_qF1 | ATATGAATTGAGACGGGAGCCT | 94 |
|  |  | rps14_Pin_qR1 | CACGAGAAACGATACGAGGAAT |  |
|  | *rps19* | rps19_Pin_qF1 | AATGAGGAGCAAGAGGGAGAAT | 89 |
|  |  | rps19_Pin_qR1 | CAGATTTATGCCCAACCTTTCC |  |
|  | *sdh3* | sdh3_Pin_qF1 | GCCGTTATCACCTCATCTTACTATTC | 91 |
|  |  | sdh3_Pin_qR1 | ATCCCACCATGAATGACGAACT |  |
|  | *sdh4* | sdh4_Pin_qF1 | CATTTGTCCCTATCTATTGGTGG | 93 |
|  |  | sdh4_Pin_qR1 | AAGAACGATCCCAGATAGATGAA |  |
| *Podocarpus macrophyllus* | *LFY* | LFYE1F3 | TGCAGCTTTCTTCAAGTGGGA | 89 |
|  |  | LFYE1R3 | TCACAGTGAAACCCATATCCAC |  |
|  | *atp1* | atp1_Pod_qF1 | CGGAACAAAGAATTACCAACTA | 93 |
|  |  | atp1_Pod_qR1 | CCCAAATACGACGATACCTACA |  |
|  | *atp4* | atp4_Pod_qF1 | AGCTCGTCGTTTCATAGGCTTTA | 93 |
|  |  | atp4_Pod_qR1 | CTGACTCCACATTTGGGTTTCG |  |
|  | *atp6* | atp6_Pod_qF1 | TTGAGCAATTCGCCATTATCCC | 99 |
|  |  | atp6_Pod_qR1 | ACCGCCTATTTGTTCGTTTACCAG |  |
|  | *atp8* | atp8_Pod_qF1 | GCAGGATTCCCAAACCACG | 92 |
|  |  | atp8_Pod_qR3 | GGGATATTTCGGATAAACTGGA |  |
|  | *atp9* | atp9_Pod_qF1 | GGGCTGCTGTCGGTATTGGA | 96 |
|  |  | atp9_Pod_qR1 | CGATGCAGTAGCTTCGGTTGG |  |
|  | *ccmB* | ccmB_Pod_qF1 | TAGGTCTGATTCGGATTCCTCC | 94 |
|  |  | ccmB_Pod_qR1 | GTAAAGATCGGGCTCCCTGGTG |  |
|  | *ccmC* | ccmC_Pod_qF1 | CTTCCTGTCGAACTGGCTCCTA | 97 |
|  |  | ccmC_Pod_qR1 | TACCAAATTGGCTTATGCTCCC |  |
|  | *ccmFC* | ccmFC_Pod_qF1 | CGGACGTAAGAGTTTCTGGTT | 94 |
|  |  | ccmFC_Pod_qR1 | CAAGGGATTCGTATGTATAGCC |  |
|  | *ccmFN* | ccmFN_Pod_qF1 | TTTCCGTTGCATTCACTTACGA | 100 |
|  |  | ccmFN_Pod_qR1 | GAAAGTTGGAGGGAGTATGACG |  |
|  | *cob* | cob_Pod_qF1 | TTCGCTATCTCCTCCTCCATTT | 93 |
|  |  | cob_Pod_qR1 | CTATTGCGGCTACACCTCCTAA |  |
|  | *cox1* | cox1_Pod_qF1 | CAGCCTTGGTAGAAGTGGGTAG | 97 |
|  |  | cox1_Pod_qR2 | CCGCGCATGTTAGAGATAGTAG |  |
|  | *cox2* | cox2_Pod_qE1F1 | TAAAGGTCCTCACAATCAAGCC | 89 |
|  |  | cox2_Pod_qE1R1 | GACCGACATCCATGATACGAAA |  |
|  | *cox3* | cox3_Pod_qF1 | ATCGTCTCGGAGGTAATGTTCT | 93 |
|  |  | cox3_Pod_qR1 | ACGGTGGCTACTAAAGCGTAAA |  |
|  | *matR* | matR_Pod_qF1 | TCGCAGCGAGCAGCTAGGTTTG | 103 |
|  |  | matR_Pod_qR2 | GCGGAGGATTTGTGCTTGTGG |  |
|  | *mttB* | mttB_Pod_qF1 | GTATGCTCCCAAGTACCCGTAA | 94 |
|  |  | mttB_Pod_qR1 | AAACAAGCGATGATTTGACACC |  |
|  | *nad1* | nad1_Pod_qE1F1 | GTAGCCTTCTTAGTGTTAGCCGA | 91 |
|  |  | nad1_Pod_qE1R1 | ACTTGGTGGAATGGGTTCTTTA |  |
|  | *nad2* | nad2_Pod_qE4F1 | ATCGCTACTAATTGGTATCTTCATC | 96 |
|  |  | nad2_Pod_qE4R1 | CGCTAGTCACTACTCCCATCAA |  |
|  | *nad3* | nad3_Pod_qF1 | CGATCCCACTTGGTGTTCCTCTC | 96 |
|  |  | nad3_Pod_qR1 | CGAAACGACTTCTGGCATCACC |  |
|  | *nad4* | nad4_Pod_qE2F1 | GATCAGGGCAGCATATCAGTTT | 92 |
|  |  | nad4_Pod_qE2R1 | TCGGTGGTTGGTAGGATTTGTA |  |
|  | *nad4L* | nad4L_Pod_qF2 | TTCGGTATTTCCCGTTTATCTG | 92 |
|  |  | nad4L_Pod_qR1 | ATGCGATTGATAGATTCTACTGC |  |
|  | *nad5* | nad5_Pod_qE2F1 | TGCCATGTCGGATGAGCAGGAT | 101 |
|  |  | nad5_Pod_qE2R1 | AGCGAACGGAAGGAGTGGTAAG |  |
|  | *nad6* | nad6_Pod_qF1 | CGGTTCTCGATTTCTAGCCCTAT | 95 |
|  |  | nad6_Pod_qR1 | TTGGTCTGTCGTCCTCCTCATT |  |
|  | *nad7* | nad7_Pod_qE4F1 | AGTGGTGTAATGCCAAGAGGTT | 100 |
|  |  | nad7_Pod_qE4R1 | CGATACGGATACAGTAGCGATC |  |
|  | *nad9* | nad9_Pod_qF1 | GCCGATACGGACTACCCATTC | 95 |
|  |  | nad9_Pod_qR1 | GATGGAAATGGACTTACTACCG |  |
|  | *rpl2* | rpl2_Pod_qF3 | TTGTGAGATTGCCGTCTGGTGC | 96 |
|  |  | rpl2_Pod_qR1 | GCAACTCCACGAACAACAGGTC |  |
|  | *rpl5* | rpl5_Pod_qF1 | TTCGTACAGGCACCACAACCAG | 94 |
|  |  | rpl5_Pod_qR1 | TCGGAAATCTCGGAATGCTCTT |  |
|  | *rpl16* | rpl16_Pod_qF1 | AAACCTACGGAAGTGAGAATGG | 92 |
|  |  | rpl16_Pod_qR1 | CAGCGAACGAACTTGGTAGACA |  |
|  | *rps1* | rps1_Pod_qF2 | AACAATAAAGTCGGCTTCGTCG | 102 |
|  |  | rps1_Pod_qR2 | CCACCATCTCTGCCAACCTCA |  |
|  | *rps2* | rps2_Pod_qF2 | CATTTTCTGGGTTCCCTCTCACG | 100 |
|  |  | rps2_Pod_qR1 | CCCGCTCAGCATTCATAACCAC |  |
|  | *rps3* | rps3_Pod_qF2 | ACCAGTTCGGGATATTCCATTA | 93 |
|  |  | rps3_Pod_qR2 | CTTTGACACCCAAGATTCCATA |  |
|  | *rps4* | rps4_Pod_qF1 | TCTCCATTCCTGCGAAACTCTT | 95 |
|  |  | rps4_Pod_qR1 | TTGCCCACGATTCTATTGACGA |  |
|  | *rps7* | rps7_Pod_qF3 | TCAAACTCTGCATCGCCTAGCT | 95 |
|  |  | rps7_Pod_qR3 | GTACATGATAAGTGGTACCTGC |  |
|  | *rps10* | rps10_Pod_qF2 | GGTTTTCGGAAGGTTGGATTG | 98 |
|  |  | rps10_Pod_qR2 | CTCTATTCATGTCTAGCCGTGTT |  |
|  | *rps11* | rps11_Pod_qF2 | GAGCTGCAAGGAACTTAGGTGT | 97 |
|  |  | rps11_Pod_qR2 | AAGCCTGCATCCATTGTGTG |  |
|  | *rps12* | rps12_Pod_qF1 | TCGGATGAATGTCCTCAGAAGC | 93 |
|  |  | rps12_Pod_qR1 | TGTTCCTGCGGATTATGACCTT |  |
|  | *rps13* | rps13_Pod_qF1 | TGAACGAATAATTGGTCGAGAT | 92 |
|  |  | rps13_Pod_qR1 | ACAAGTCCCAGCATTAGTATGG |  |
|  | *rps14* | rps14_Pod_qF2 | TATGAACTGAAACGGATGCTCT | 100 |
|  |  | rps14_Pod_qR3 | GGATGCTAATTCTCGGAAAACTA |  |
|  | *rps19* | rps19_Pod_qF1 | GTTTTGTCGATGCCTTCCTGTC | 96 |
|  |  | rps19_Pod_qR1 | CGGATTTATGACCAACCTTTCC |  |
|  | *sdh3* | sdh3_Pod_qF2 | CGTTACCACCTCATCCCACTAT | 96 |
|  |  | sdh3_Pod_qR2 | ATCCCACCATAAATGACGAACT |  |
|  | *sdh4* | sdh4_Pod_qF2 | GTGGTTCGGTTATTCCTATTTG | 95 |
|  |  | sdh4_Pod_qR2 | GATCCCCACATGGATATGC |  |
| *Araucaria cunninghamii* | *LFY* | LFYE1F3 | TGCAGCTTTCTTCAAGTGGGA | 95 |
|  |  | LFY_Ara_E1R1 | GTGAAACCCATCTCAAACATCTT |  |
|  | *nad5* | nad5_Ara_qE2F1 | GCCATGTCGGATGAGCAAGATA | 106 |
|  |  | nad5_Ara_qE2R1 | GTCAGAAAGAGCGAACGGAAAG |  |
|  | *rpl2* | rpl2_Ara_qF4 | TTCAAGCCGATCGTGCGCTCGT | 95 |
|  |  | rpl2_Ara_qR5 | TGAATCCACGTGCCCAGCCGCAT |  |
|  | *rps1* | rps1_Ara_qF1 | CGAGGTTAAACAAGGCGGGAT | 105 |
|  |  | rps1_Ara_qR1 | ATTTCTGCGGCGGAATTATGTG |  |
|  | *rps2* | rps2_Ara_qF2 | CTACTGAACACGAACGCCCACC | 98 |
|  |  | rps2_Ara_qR3 | AGGGTCGCCCACTGAGTGTCGT |  |
|  | *rps7* | rps7_Ara_qF1 | TTATCAAACCCTCCAGCGTCTA | 103 |
|  |  | rps7_Ara_qR1 | CTTTCTTTCGGTAAGCATCCAG |  |
|  | *rps10* | rps10_Ara_qF1 | GTCGGATTGCCTAAGAGGAGAA | 106 |
|  |  | rps10_Ara_qF2 | GCCAAGATACGGATAGTGATG |  |
|  | *rps11* | rps11_Ara_qF1 | GAGTATGAGCATTGGGAACGAC | 109 |
|  |  | rps11_Ara_qR1 | CTTCAAGCAACTACCAGCAGAG |  |
|  | *rps14* | rps14_Ara_qF1 | ATCCCAACCTTCCTAGTGATTT | 103 |
|  |  | rps14_Ara_qR1 | TACAAAGACGCTTTCCTTATGC |  |
|  | *sdh3* | sdh3_Ara_qF2 | TACCAATACCTACCAATCCCTCT | 101 |
|  |  | sdh3_Ara_qR2 | AATCCCACCATAAATGACGAAC |  |
| *Sciadopitys verticillata* | *LFY* | LFY_E1F3 | TGCAGCTTTCTTCAAGTGGGA | 99 |
|  |  | LFY_Sci_E1R2 | ACAGTTCCTCTAGGCAGCTCA |  |
|  | *nad5* | nad5_Sci_qE2F1 | GCTTTCTGGTTGGGAAGTGTCT | 101 |
|  |  | nad5_Sci_qE2R2 | GTCTCGCCCGAATGGATTAGTT |  |
|  | *rpl2* | rpl2_Sci_qF1 | GCATTCAGCAGGTATCTCCACT | 102 |
|  |  | rpl2_Sci_qR1 | AGCACCTGATGGTAATCGCACA |  |
|  | *rps1* | rps1_Sci_qF1 | CTAACCAGAGTTCCCGCTAATC | 102 |
|  |  | rps1_Sci_qR1 | CCGTTTCGTTCAGTCTCATAGC |  |
|  | *rps2* | rps2_Sci_qF1 | GACGAGATAACGGAACAAACGG | 103 |
|  |  | rps2_Sci_qR1 | CTCTGCATTCATTACCACCACA |  |
|  | *rps7* | rps7_Sci_qF1 | GATCCAGGCTTAGAGCAGAAAC | 96 |
|  |  | rps7_Sci_qR1 | ACTCGCACTTTCGACACTTCAC |  |
|  | *rps10* | rps10_Sci_qF2 | ATCGGATCGGCGACGCCAAGAT | 106 |
|  |  | rps10_Sci_qR2 | GAGACGCAGGCGTTTGAACCAA |  |
|  | *rps11* | rps11_Sci_qF5 | GCTGATTTCCTTGCTCTCAGCA | 96 |
|  |  | rps11_Sci_qR2 | CCTTATCATCTGTCACAGTAAC |  |
|  | *rps14* | rps14_Sci_qF1 | CAAGCGAAACATACAAGATCAC | 108 |
|  |  | rps14_Sci_qR1 | CTTACACCCATTAACTCACC |  |
|  | *sdh3* | sdh3_Sci_qF1 | TCAATCGTCCGTTATCACCTCA | 101 |
|  |  | sdh3_Sci_qR1 | TAGTGGGCCTATCTCCAAGCA |  |
| *Cunninghamia lanceolata* | *LFY* | LFYE1F3 | TGCAGCTTTCTTCAAGTGGGA | 108 |
|  |  | LFYE1R1 | GCATTCTCATCAATCTTGCGT |  |
|  | *nad5* | nad5_Cun_qE2F1 | ATTACAGAACGATCTGAAGAGG | 104 |
|  |  | nad5_Cun_qE2R1 | ATAGGTCAAGGGTAACGAGGAA |  |
|  | *rpl2* | rpl2_Met_qF1 | TGCCGAGCCACTATTGGTATGG | 105 |
|  |  | rpl2_Met_qR1 | TTGTGCGTCCTTCACCTCCTC |  |
|  | *rps1* | rps1_Met_qF1 | AGGCAGCATAGGGCTTGGATC | 112 |
|  |  | rps1_Met_qR1 | CACGGCATAACCTCTGTTCACT |  |
|  | *rps2* | rps2_Cun_qF3 | CGATGAGATAACGGAACAGACT | 113 |
|  |  | rps2_Cun_qR4 | TGCCGCCTGCTTTCCTCGTAT |  |
|  | *rps7* | rps7_Cun_qF1 | AGCTCTTCGTTGGCTCCTTG | 103 |
|  |  | rps7_Cun_qR1 | TCTTTCGGTAAGCATCCAGTA |  |
|  | *rps10* | rps10_Cun_qF1 | GAAGAAATACCTGAACCACCCG | 102 |
|  |  | rps10_Cun_qR1 | GGATCGAAGCACCGTATAAAGC |  |
|  | *rps11* | rps11_Met_qF1 | GAAGATTATGAGCAGTGGGAGC | 102 |
|  |  | rps11_Tax_qR3 | AACAGCATACTTGGTCCTACGG |  |
|  | *rps14* | rps14_Cun_qF1 | GCCAAGAAATAGTTCCTTCACA | 95 |
|  |  | rps14_Cun_qR1 | TACACCCATCAACTCACCTTTA |  |
|  | *sdh3* | sdh3_Cun_qF1 | TTACGAGCCACAACTTACTTCA | 97 |
|  |  | sdh3_Cun_qR1 | CAAATCCCACCATAAATGACGA |  |
| *Metasequoia glyptostroboides* | *LFY* | LFYE1R1 | GCATTCTCATCAATCTTGCGT | 104 |
|  |  | LFYE1F1_Met | GTCAATATGACTGAACAAGAGCT |  |
|  | *nad5* | nad5_Cun_qE2F1 | ATTACAGAACGATCTGAAGAGG | 101 |
|  |  | nad5_Cun_qE2R1 | ATAGGTCAAGGGTAACGAGGAA |  |
|  | *rpl2* | rpl2_Met_qF1 | TGCCGAGCCACTATTGGTATGG | 97 |
|  |  | rpl2_Met_qR1 | TTGTGCGTCCTTCACCTCCTC |  |
|  | *rps1* | rps1_Met_qF1 | AGGCAGCATAGGGCTTGGATC | 101 |
|  |  | rps1_Met_qR1 | CACGGCATAACCTCTGTTCACT |  |
|  | *rps2* | rps2_Met_qF1 | CGGAACATACTGCCGCTAAACT | 99 |
|  |  | rps2_Met_qR1 | TATAATGGATCTACCGCCTGCT |  |
|  | *rps7* | rps7_Met_qF1 | GCGCAAACAGATAGTGCAACCA | 98 |
|  |  | rps7_Met_qR1 | CCAGTACCTCGGCAGATAAACA |  |
|  | *rps10* | rps10_Met_qF3 | TGAACCATCCGTGCTTCCAGAG | 107 |
|  |  | rps10_Met_qR3 | TGCGCTTAAACCAGAAGAACAT |  |
|  | *rps11* | rps11_Met_qF1 | GAAGATTATGAGCAGTGGGAGC | 112 |
|  |  | rps11_Tax_qR3 | AACAGCATACTTGGTCCTACGG |  |
|  | *rps14* | rps14_Met_qF3 | TACGAGATCACAAGCGCAGATTG | 91 |
|  |  | rps14_Met_qR3 | AGACGGCACGAGAACGACCAGT |  |
| *Welwitschia mirabilis* | *LFY* | LFY_E1F3 | TGCAGCTTTCTTCAAGTGGGA | 97 |
|  |  | LFY_Wel_E1R1 | CACAGTAAAGCCCATCTCCATC |  |
|  | *nad2* | nad2_Wel_qF1 | GATACACCGAGAACATGGATGA | 97 |
|  |  | nad2_Wel_qR1 | AAGGGAGAAGGATACAGGAAGA |  |
|  | *rps1* | rps1_Wel_qF4 | GCAAGATTGTTCGCAAAGAGTA | 101 |
|  |  | rps1_Wel_qR4 | ATTCCTCGAGCCGTCTGTAGCT |  |
|  | *rps10* | rps10_Wel_qF1 | GTCAATCAACATCGGTCCATAC | 90 |
|  |  | rps10_Wel_qR1 | CATTCGCATGGGCGTAGTTTC |  |
|  | *rps11* | rps11_Wel_qF1 | CAACAGTTTCTGCTGGTTGCTT | 98 |
|  |  | rps11_Wel_qR1 | TTATTGGCCTCCCTCTATTTCG |  |
|  | *rps14* | rps14_Wel_qF1 | GATCATTACCGCAGACTTCTTG | 96 |
|  |  | rps14_Wel_qR1 | TCTTATCCGTGTCAAGGAACTG |  |
| *Gnetum montanum* | *LFY* | LFY_E1F3 | TGCAGCTTTCTTCAAGTGGGA | 98 |
|  |  | LFY_Gne_qR1 | AACCCATCTCCATCATCTTAGTCA |  |
|  | *atp1* | atp1_Gne_qF1 | CTTCGGATTCTGCTCCTCTTCA | 98 |
|  |  | atp1_Gne_qR1 | GACCGTTTAGCCGCTCTTTCTA |  |
|  | *atp4* | atp4_Gne_qF1 | TTCAGTCAGAGGAGTTTGGGT | 98 |
|  |  | atp4_Gne_qR1 | TCCACATTTAGGTTTCGGCATA |  |
|  | *atp6* | atp6_Gne_qF1 | TACCACTGCCGTTAGCACCTTT | 98 |
|  |  | atp6_Gne_qR1 | ATAGCATAGTCCAGGCGAACC |  |
|  | *atp8* | atp8_Gne_qF1 | TGGAGAAATACGTGGCTCAC | 98 |
|  |  | atp8_Gne_qR1 | CCAGATAGAGGATTGGGATAAG |  |
|  | *atp9* | atp9_Gne_qF1 | GAGCTGCCGTTGGTATTGGA | 97 |
|  |  | atp9_Gne_qR1 | CGAATAAGATAAGAAACGCCATC |  |
|  | *ccmB* | ccmB_Gne_qF1 | TTCGCAGTGATAAAGAAGACGG | 97 |
|  |  | ccmB_Gne_qR1 | AGCCGAACGAGAATGAATACCA |  |
|  | *ccmC* | ccmC_Gne_qF1 | GCATTAGCCAATTTGGTACATC | 97 |
|  |  | ccmC_Gne_qR1 | GAGCTTCCATTTCTTCCGTTA |  |
|  | *ccmFC* | ccmFC_Gne_qF1 | CCATTCAAGCGAGCACAACTAA | 96 |
|  |  | ccmFC_Gne_qR1 | CCTTCTGGAGCTGACGTAACA |  |
|  | *ccmFN* | ccmFN_Gne_qF1 | CTCACAGACAAATGCCATCCCT | 98 |
|  |  | ccmFN_Gne_qR1 | CTAGCAAGATGCCTACGGTGA |  |
|  | *cob* | cob_Gne_qF1 | ATTCCTCGCAGTATCCCAGACA | 96 |
|  |  | cob_Gne_qR1 | GGTTCACATCCGATCCAACCTA |  |
|  | *cox1* | cox1_Gne_qF1 | TCAGCCTTGGTAGAAGTGGGTA | 96 |
|  |  | cox1_Gne_qR1 | CCGCGCATGTTGAAGATAGTAG |  |
|  | *cox2* | cox2_Gne_qF1 | CGGCAACACCAGTGATGCAAG | 99 |
|  |  | cox2_Gne_qR1 | GCTGGATTTACCACTACCTCGTC |  |
|  | *cox3* | cox3_Gne_qF1 | GCTGCCGTAACTTGGGCTCATC | 97 |
|  |  | cox3_Gne_qR1 | CGAGATAGTGAAAGGTGCTTGATA |  |
|  | *matR* | matR_Gne_qF1 | AGCTGGTTATCGGATTAGAAAG | 97 |
|  |  | matR_Gne_qR1 | AGTGGTAAGTCGTCGGCATA |  |
|  | *mttB* | mttB_Gne_qF1 | AGTTGCTACGGAGAACGAAGG | 94 |
|  |  | mttB_Gne_qR1 | TACCCGCAAAGCATAGGAAGTG |  |
|  | *nad1* | nad1_Pod_qE1F1 | GTAGCCTTCTTAGTGTTAGCCGA | 97 |
|  |  | nad1_Gne_qE1R1 | CCCAAGCAACCAAACTTAGCAT |  |
|  | *nad2* | nad2_Gne_qE4F1 | TCATGTGGAACCATAGGAGGCA | 96 |
|  |  | nad2_Gne_qE4R1 | CAAAGCGGCGGAGAACAAATAG |  |
|  | *nad3* | nad3_Gne_qF1 | TCGGTGATGCCAGAAGTCGTT | 96 |
|  |  | nad3_Gne_qR1 | TACTCCCAATCTAAAGCACCCT |  |
|  | *nad4L* | nad4L_Gne_qF1 | ACTTCTCGGTATTTCCCGTTTA | 96 |
|  |  | nad4L_Gne_qR1 | ATTCTACTGCAATGGTCCCTC |  |
|  | *nad5* | nad5_Pin_qF1 | GGGCTTGCTTCCTCGTTACCTT | 100 |
|  |  | nad5_Gne_qR1 | GTAAGATGTCCCGCCCAAATGA |  |
|  | *nad6* | nad6_Gne_qF1 | GCAACACCTCGGGTTTACTTAT | 97 |
|  |  | nad6_Gne_qR1 | TCGGTCCAATAATACCACTCAC |  |
|  | *nad9* | nad9_Pin_qF1 | CCGATACGGACTACCCATTTCA | 90 |
|  |  | nad9_Pin_qR1 | TCCCATACTTCTCGCTCCCACC |  |
|  | *rpl16* | rpl16_Pin_qF1 | AAACCTACAGAAGTGAGAATGG | 95 |
|  |  | rpl16_Gne_qR1 | CGAGCATCTGACAAACTCAC |  |
|  | *rps2* | rps2_Gne_qF2 | GCTCTTGTTGATTCGGAAACCT | 100 |
|  |  | rps2_Gne_qR2 | CATCTGCTTCCAGCATTGGGT |  |
|  | *rps3* | rps3_Gne_qF1 | CGCTAAAGCCGTCGTAGTTCCA | 99 |
|  |  | rps3_Gne_qR1 | CTCGCTCGCATCACATAACCCT |  |
|  | *rps4* | rps4_Gne_qF1 | AAGCCATACGAAAGTTGTCCCT | 98 |
|  |  | rps4_Gne_qR1 | CGCTTGAGGAATAGTTTCACAG |  |
|  | *rps10* | rps10_Gne_qF1 | GTATTGCGGTCACCTCACATTG | 95 |
|  |  | rps10_Gne_qR1 | AGTCTTCATCGGCAGTCGTAG |  |
|  | *rps11* | rps11_Gne_qF2 | TCATCAGATCAGGAATGCCCAT | 98 |
|  |  | rps11_Gne_qR1 | ATTTGGACCTCAGACGACCTAC |  |
|  | *rps12* | rps12_Gne_qF1 | TTGGAGAAATGTCCTCAGAAGC | 98 |
|  |  | rps12_Gne_qR1 | TGTAAGCAAATAGCTCGTGTCG |  |
|  | *rps13* | rps13_Gne_qF2 | CGATTAGGTATCAGTGGGAACA | 94 |
|  |  | rps13_Pin_qR1 | GCGACAAGTCCTAGCATTAGTATG |  |
|  | *rps14* | rps14_Gne_qF2 | GATCACTATCGCAGACTTCTTG | 104 |
|  |  | rps14_Gne_qR2 | TTCTCAGTTTCAAACGAGCATC |  |
|  | *sdh3* | sdh3_Gne_qR2 | TCAGGCTTGTCTGCCCTATTTC | 105 |
|  |  | sdh3_Gne_qR1 | AAGAACTTTGCTCCTGCCCTCC |  |
| *Ephedra przewalskii* | *LFY* | LFY_Eph_E2F1 | GACCCTGTTCTATTCCTCCAAA | 102 |
|  |  | LFY_Eph_E2R1 | CCAGGCTCTGTGACTATGAAGG |  |
|  | *nad5* | nad5_Eph_E2F1 | TGATCTTTGCTTGCGGCATTTC | 101 |
|  |  | nad5_Eph_E2R1 | ATGGCATAGGTGAAAGGTAACGAGAA |  |
|  | *rps2* | rps2_Eph_F1 | CTCCACTTCGCCAACAGCAACC | 106 |
|  |  | rps2_Eph_R1 | TTGAGGGTGACGATGGCGTCG |  |
|  | *rps10* | rps10_Eph_F1 | TCGCACGGGATAACGAAGAG | 98 |
|  |  | rps10_Eph_R1 | AGGAATACCTAGCATTGATGAGC |  |
|  | *rps11* | rps11_Eph_F2 | AGTTCTGAATAAGGAGGTGAGGC | 107 |
|  |  | rps11_Eph_R2 | TTCTAAGTCGGCAACCTCCAT |  |
|  | *rps14* | rps14_Eph_F1 | AGAAAGGCGCAACCGTCAAGAT | 104 |
|  |  | rps14_Eph_R1 | CGAGCAAGCTCACGTAGAACAA |  |
|  | *sdh3* | sdh3_Eph_F1 | CTCTTCAAGCCTCACTTTCTAC | 102 |
|  |  | sdh3_Eph_R1 | AATGCTTCTTTGAGTCTTTAAGA |  |
| **identification of the spliced mode of intron** | | | | |
| *Cephalotaxus sinensis* | nad2i542 | nad2_CepE1F1 | GATTATCCGCCGTTAGCAAGTA |  |
|  |  | nad2_CepE2R1 | GAGCACCGAACAAAGTCATTTC |  |
|  | nad2i1282 | nad2_CepE3F1 | ATGTGGAACCATAGGAGGAATT |  |
|  |  | nad2_CepE4R1 | AGTAACTAAGAACGAGGGAGGG |  |
|  | nad4i461 | nad4_CepE1F2 | GGTTGGTCCAGTATAGAAAGTTATG |  |
|  |  | nad4_CepE2R2 | AGAACAGATCCGAGTGAAGTGTAT |  |
|  | nad4i976 | nad4_CepE2F2 | GTAGCCCATACGAATCCTGTGA |  |
|  |  | nad4_CepE3R2 | GGATTCGGCATGGTGCTTACTG |  |
|  | nad4i1399 | nad4_CepE3F2 | TCACCTGGTACTAGCAGCTCTATC |  |
|  |  | nad4_CepE4R2 | CATGTTGCACTAAGTTACCTACGG |  |
|  | nad5i230 | nad5_CepE1F1 | ATCGTTGCCCTTGCTCGGTAGT |  |
|  |  | nad5_CepE2R1 | GCTTTATCCGCCTGAAGTCGTG |  |
|  | nad5i1455 | nad5_CepE2F1 | TCTCACTACTCTTTCCGTTCGC |  |
|  |  | nad5_CepE3R1 | TACCTAAACCAATCATCATATC |  |
|  | nad5i1477 | nad5_CepE3F1 | GATATGATGATTGGTTTAGGTA |  |
|  |  | nad5_CepE4R1 | ATCTCAATAGCACCTTTGTCTG |  |
| **verification of missing genes** |  |  |  |  |
|  | *rpl10* | rpl10_gymF1 | ATTCCAGTGGCTTGACCAGT |  |
|  |  | rpl10_gymR1 | AAGTGGGACCTGCACTATGA |  |
|  |  | rpl10_gymF2 | AGTGGCTCGACCAGTAACCAATG |  |
|  |  | rpl10_gymR2 | GAACTGGGTAAATGGAAATGG |  |
|  |  | F1* | ATGCCATTCGGAAGAAGTMT |  |
|  |  | R148* | GGAACACACGAAASAAAGATATRAAC |  |
|  |  | R159* | TTAGGTGGTATYCCGAGATYGA |  |

* Mower JP, Bonen L. 2009 Ribosomal protein L10 is encoded in the mitochondrial genome of many land plants and green algae. *BMC Evol. Biol.* **9**, 265. (doi:10.1186/1471-2148-9-265).
